# Exploring the omnigenic architecture of selected complex traits

**DOI:** 10.1016/j.ajhg.2025.07.006

**Published:** 2025-08-04

**Authors:** Florin Ratajczak, Matthias Heinig, Pascal Falter-Braun

**Affiliations:** 1 Institute of Network Biology (INET), Molecular Targets and Therapeutics Center (MTTC), Helmholtz Center Munich, German Research Center for Environmental Health, Munich-Neuherberg, Germany; 2 Institute of Computational Biology (ICB), Helmholtz Center Munich, German Research Center for Environmental Health, Munich-Neuherberg, Germany; 3 Department of Computer Science, TUM School of Computation, Information and Technology, Technical University of Munich, Garching, Germany; 4 German Centre for Cardiovascular Research (DZHK), Munich Heart Association, Partner Site Munich, Berlin, Germany; 5 Microbe-Host Interactions, Faculty of Biology, Ludwig-Maximilians-Universität (LMU) München, Planegg-Martinsried, Germany

**Keywords:** omnigenic model, core genes, GWAS, graph neural networks, ulcerative colitis, schizophrenia, coronary artery disease

## Abstract

Genome-wide association studies (GWASs) have statistically identified thousands of loci influencing a trait of interest. To explain the organizational principles among the functionally often unrelated encoded proteins, the omnigenic model postulates core genes with direct and peripheral genes with indirect effects on molecular trait etiology. However, both core genes and the network paths by which they are influenced are unknown for most traits. Using our previously developed Speos framework to identify core genes, we here focus on the autoimmune disease ulcerative colitis (UC) to explore the regulatory relationships between core and peripheral genes and their organization in multi-modal molecular networks. The identified core genes are characterized by tissue-specific expression and trait-relevant network connections. Using genome-scale perturbation data, we demonstrate that one-third of overexpression or knockdown perturbations impact core genes differently than peripheral genes, a pattern that is not observed for GWAS or random genes. This coordinated perturbation response by core genes was robust across traits and cell lines, despite differing causal perturbagens, suggesting a universal core-gene property. Intriguingly, co-perturbation simulations suggest frequent genetic interactions between core genes, highlighting the role of non-additive interactions previously not considered in the omnigenic model. Thus, physiologically relevant core-gene sets occupy a central position in the underlying molecular network, resulting in genome-wide coordinated regulation. As previous theoretical studies have shown that coordinated regulation of core genes could explain much of the missing heritability, our qualitative observation can provide a foundation for detailed quantitative analyses.

## Teaser

Core genes identified for ulcerative colitis, coronary artery disease, and other traits by a multi-modal graph machine-learning pipeline are expressed in disease-relevant tissues. Core genes demonstrate concerted up- and downregulation upon perturbation, indicating shared functions. Co-perturbation of core genes leads to non-linear interactions, suggesting an additional layer of complexity of the omnigenic model.

## Introduction

Predicting physiologic variation by combining genetics and the constantly growing trove of systems-level data for diverse molecular modalities is at the heart of 21st century biology, with vast implications in fields ranging from agriculture to medicine. Over the past decade, genome-wide association studies (GWASs) have made significant progress linking phenotypic outcomes to specific single-nucleotide polymorphisms (SNPs) that mark genomic loci with relevant variation. A key finding that emerged from these data is that most genetic associations cannot readily be linked to the phenotype mechanistically.^1^ Part of the challenge is the accurate mapping of genetic marker variants to genes. Here, common heuristics such as assigning each variant to the closest gene or more recently developed integrative approaches are effective in practice.^1^^,^^2^^,^^3^^,^^4^^,^^5^^,^^6^ However, even after assigning trait-associated variants to genes, the mechanisms by which the genes exert their effect on a trait are often unclear.

**Figure 1:**
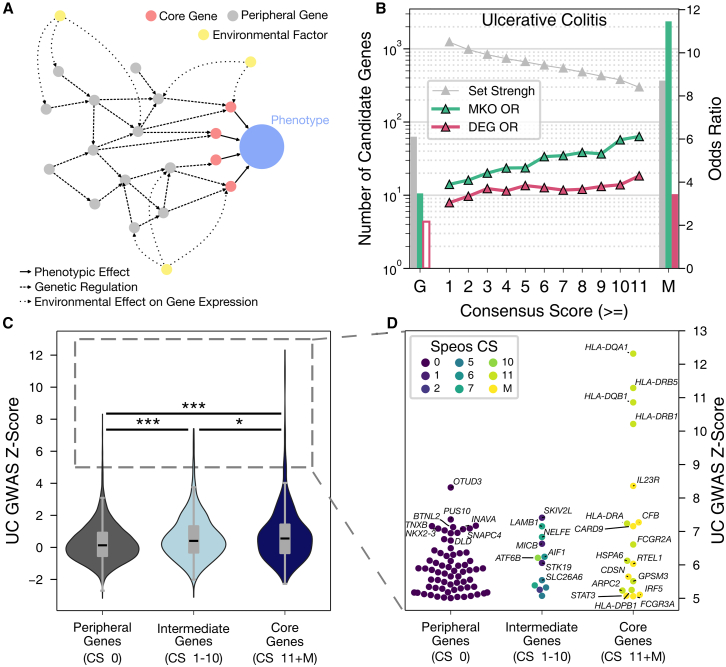
Overview of the omnigenic model and identified core genes for UC (A) Schematic overview of the omnigenic model. (B) Set strengths (gray, left *y* axis) of GWAS genes (G), Speos candidates (consensus score 1–11), and OMIM-derived genes (M). Teal and red indicate odds ratios (OR, right *y* axis) of mouse knockout (MKO) and differentially expressed genes (DEG) among the indicated sets, respectively. Markers and filled bars indicate a significant enrichment compared to peripheral genes (FDR < 0.05; for individual *p* values see Tables S4 and S5). (C and D) UC GWAS *Z* score of peripheral genes, ambivalent genes, and core genes, and individual genes with *Z* score >5. ∗*p* value < 0.05 (two-sided t test), ∗∗∗*p* value < 0.001 (two-sided t test)

Considering the interconnectedness of genes and their encoded protein and RNA products in multiple layers of biological networks, the omnigenic model aims to bridge this gap^7^ by suggesting that only a small subset of genes, the core genes, have a direct effect on a given trait, whereas the remaining majority, so-called peripheral genes, influence the trait indirectly via modulation of core-gene expression and function in disease-relevant tissues^7^^,^^8^^,^^9^^,^^10^ (Figure 1A). The core-gene model makes specific predictions for the properties of core genes, which are expected to be loss-of-function intolerant and expressed in disease-relevant tissues. As functional mutations of core genes can have strong effects on a trait,^8^ Mendelian disorder genes, where individual mutations can suffice for disease development, are obvious examples of strong core genes.^11^ Naturally, the impact of individual core genes on disease manifestation and heritability varies, e.g., depending on the complexity of the trait and the spectrum of variants in the population. However, even when the omnigenic model is applied to simple traits, such as levels of a metabolite, genetic variations at core genes cumulatively only account for 30% of the heritability despite their significant individual effects.^6^ Consequently, 70% of heritability remains to be explained by peripheral genes and the regulatory and biochemical connections that mediate their influence on core genes and their encoded proteins.^6^ A foundational assumption of the omnigenic model is that sets of peripheral genes concertedly influence numerous core genes and thereby can have a strong individual effect on the phenotype.^8^ The specific biochemical and regulatory paths within molecular networks by which peripheral genes transmit their influence on core genes can be considered as the “omnigenic architecture.”

Unraveling the omnigenic architecture of a trait requires not only knowledge of the specific core genes but also identification of which peripheral genes impact them through which network paths in disease-relevant tissues and cell types. If the network paths and their condition-specific functioning were known, this would not only illuminate how small, coordinated regulatory effects of peripheral genes converge on relevant core genes to influence the phenotype but would, conversely, also enable the prediction of the most impactful peripheral genes once the core genes are known. However, while the omnigenic model is a helpful theoretical framework to approach complex diseases, our understanding of information processing by complex networks is still too rudimentary, and consequently the specific omnigenic network architectures of complex traits remain unknown. In the absence of comprehensive mechanistic models, demonstration of whether the postulated converging regulatory effects of peripheral genes on core genes exist will be key toward supporting the omnigenic model and furthering our understanding of the dynamic organization of biological systems. Addressing this question requires knowledge of core and peripheral genes for representative complex traits.

We recently developed a graph machine-learning framework, Speos, to identify core genes from GWASs^12^ and gene-expression data using multi-modal gene/protein networks.^13^ Using Mendelian disorder genes as positive examples^11^ during training, Speos learns the biochemical and regulatory context of disease-associated genes in state-of-the-art network maps describing physical and functional interactions to predict additional core genes. An ensemble strategy, in which different models are trained on overlapping Mendelian disorder gene subsets, enables the calculation of consensus scores (CSs: with discrete values from 0 to 11). The core genes identified by Speos exhibit core-gene characteristics that have been postulated by the omnigenic model.^13^ These include intolerance to functional mutations and differential gene expression in disease-affected individuals. More importantly, the identified core-gene candidates exhibit disease-related phenotypes in mouse knockout lines at higher rates than GWAS candidates.^13^ Here, we extend this study and use the core-gene predictions to explore the omnigenic architecture of an increasingly widespread complex disease, namely ulcerative colitis (UC), and probe the existence of the postulated “peripheral master regulators,” which are peripheral genes exerting a sizable effect on the phenotype by concertedly influencing several core genes with shared direction of effect.^9^ Since these genes are assumed to have a strong GWAS signal,^9^ we refer to them more generally as “highly (GWAS-)significant peripheral genes” (HSPs) to gain insights into the omnigenic architecture that links these HSPs to core genes.

We focus on the autoimmune disease UC, for which pooled twin studies estimate a heritability of 0.67, only 40% of which, i.e., 0.27, can be explained by the significantly associated GWAS SNPs.^14^^,^^15^ This leaves a large gap of unexplained genetic influence, which may possibly be encoded in the trait’s omnigenic architecture. UC is an inflammatory bowel disease characterized by a dysregulated immune response, which, potentially after an initial infection by pathogenic bacteria, is increasingly aimed toward commensal bacteria and the body’s own tissue.^16^^,^^17^ This process is accompanied by increased pro-inflammatory signaling by nuclear factor κB (NF-κB),^18^^,^^19^ tumor necrosis factor α (TNF-α),^20^ and other cytokines,^21^^,^^22^ as well as an increased tissue infiltration of lymphocytes.^23^ We aimed to leverage the ability to confidently identify core genes to explore the omnigenic architecture of this complex disease across the genome.

We show that Speos identifies UC core genes that exhibit disease-relevant gene-expression signatures, which separate them from peripheral genes with similar GWAS signals. Leveraging interpretable machine-learning techniques, we illuminate subnetworks that may biochemically mediate the genetic effects in disease-relevant tissues. In genome-scale perturbation datasets we find evidence that an unexpectedly large fraction of peripheral genes exerts significant concerted regulatory effects on core genes, which lends empirical support to one of the omnigenic model’s key predictions. Moreover, simulations indicate that joint perturbations of UC core genes frequently lead to genetic interactions either attenuating or compounding their individual effects, a finding previously not considered in the omnigenic model. Together with the ubiquitous concerted regulation of core-gene sets through peripheral genes, this could enable a large part of the genome to influence disease in unforeseen ways. Thus, our analysis of a complex disease trait extends the omnigenic model while simultaneously providing evidence for several of its central predictions and raising important questions for subsequent research.

## Methods

### Input data

Molecular interactions can be represented as network graphs. We define a network graph G as a set of edges E and nodes, or vertices, V so that G={V,E}. Furthermore, we define a set of edge types R so that each edge e∈E is assigned an edge type r∈R. The set of nodes V contains all human protein-coding genes, identified by their HGNC symbol. Edges are obtained from several sources and are typed depending on their source and cell type or tissue: HuRI,^24^ BioPlex 3.0,^25^ Recon3D,^26^ and GRNdb.^27^ Networks sourced from GRNdb represent transcriptional regulation in 27 healthy human tissues. We have selected networks that model three different kinds of physiologic interaction: direct and indirect protein-protein interactions (PPIs) (HuRI^24^ and Bioplex^25^), genetic regulation via transcription factors and enhancers (GRNdb^27^), and metabolic interactions (Recon3D^26^), such as shared metabolites. PPIs are undirected, which we modeled as two directed edges running in opposite directions between the two interacting proteins, while the other edges are directed. Gene-regulatory edges point from the transcription factors to the target genes, while metabolic edges point from a gene whose protein partakes in a reaction that produces a metabolite to the gene whose protein uses the metabolite as educt.

Input features for every node can be separated into GWAS sourced and gene expression. GWAS summary statistics are aggregated to gene-level *Z* scores using MAGMA.^28^ Individual GWAS used, sample sizes, and case numbers can be found in Table S1. Gene-expression features for 53 tissues are sourced from GTEx^29^ v.7, and an additional set of features for 18 blood cell types and total peripheral mononuclear blood cells (PBMC) is sourced from the human blood atlas^30^ v.19, resulting in a vector of 72 input features for every gene. Genes for which at least one of the mentioned input features could not be gathered are removed from V, and all adjacent edges are removed from E. The resulting total number of nodes for every trait can be found in Table S1.

### Model training and validation

Speos^13^ is an ensemble framework for positive-unlabeled learning specialized in genetic networks. It utilizes a nested cross-validation of m outer folds and n=m−1 inner folds, repeatedly holding out different parts of the rare positively labeled genes to determine whether unlabeled genes receive predictions significantly higher than random expectation. If so, these genes receive one CS point from this fold up to a CS of *m*, where *m* is the number of outer folds. Therefore, Speos assigns a CS to every unlabeled gene as a measure of certainty. We have trained a Speos^13^ cross-validation ensemble with 11 outer and 10 inner folds using the input data described above. Using a 10-fold inner cross-validation has been shown to achieve a desirable granularity of CSs while retaining sufficiently sized holdout sets.^13^ Each model consists of two fully connected layers, two feature-wise linear modulation (FiLM)^31^ graph convolution layers interspersed with instance normalization layers,^32^ and two additional fully connected output layers. We used 50 hidden dimensions across the entire architecture and exponential linear unit (ELU)^33^ non-linearity layers after every trainable layer. This architecture has demonstrated optimal results in similar settings.^13^ Speos adaptively adjusts the epochs using early stopping on the f1 score of recall and precision on the validation holdout set. It further uses an adapted version of binary cross-entropy (BCE) loss L, taking into account the label uncertainty of the unlabeled set:

$$L=\sum_{u=0}^{u*}\frac{\text{BCE}(y_{u},{\hat{y}}_{u})}{d}+a\sum_{u=0}^{\left|P_{\text{train}}\right|}\text{BCE}(y_{p},{\hat{y}}_{p})\text{,}$$

where d is the dilution parameter set to 10, a is the amplification parameter set to 2, $\left|P_{\text{train}}\right|$ is the positive training set, and *u*^∗^ is a subset of all unlabeled genes obtained by sampling $|P_{\text{train}}|\cdot d$ unlabeled genes. We optimize L using the Adam optimizer with learning rate 10^−3^.

We obtained positive labels from OMIM^34^ using standardized clinical phenotype terms^12^ (Table S2). For validation, we have queried the Mouse Genome Informatics^35^^,^^36^ (MGI) database using the same standardized clinical phenotype terms and then compared enrichment among candidates and peripherals using Fisher’s exact tests. We use false discovery rate (FDR) adjustment for multiple testing correction of *p* values. For additional validation, we gathered differentially expressed genes from the literature and compared enrichment among candidates and peripherals using Fisher’s exact tests. Edge and input feature importance scores have been obtained using integrated gradients^37^ via the Captum^38^ interface of PyTorch Geometric.^39^ Each model’s attributions are minmax scaled into the interval [−1, 1] for input importance and [0, 1] for edge importance, then averaged across the ensemble.

### Gene set enrichment analyses

We performed gene set enrichment analyses using the Gene Ontology^40^^,^^41^ biological process and WikiPathways^42^^,^^43^ pathways. For these analyses, we use all genes included in Speos (Tables S1 and S3) as background set (universe) and test the enrichment of individual ontology terms among our core-gene set (CS11 and OMIM-derived genes) using a hypergeometric test. We finally adjust the *p* values using FDR.

### Gene classifications

Speos predicts candidate genes with increasing confidence from CS 1 to CS 11, while genes with CS 0 are not considered candidates. To increase the contrast between the two sets, we consider genes with CS 11 and the OMIM-derived genes used for training as core genes and genes with CS 0 as peripheral genes. Genes with CS 1 to CS 10 are excluded from the analyses. Individual CSs of every gene for every trait can be found in Table S3. The model-based interpretation of HSPs in the first half of this article requires us first to define HSPs within the model’s input space, i.e., by MAGMA *Z* score. Here, we define HSPs as all genes that receive a CS of 0 and have a GWAS *Z* score greater than 5. In the latter half of the article, we are not bound to the model input space and therefore use a more conventional definition of HSPs. Here, we define HSPs as genes located less than 10 kb up- or downstream of GWAS SNPs with an individual *p* value $5\times 10^{-8}$ or lower. To avoid genetic correlates, we further discard SNPs that reside less than 10 kb up- or downstream of any gene with Speos CS greater than 0 or in the same linkage disequilibrium (LD) block as OMIM-derived genes or core genes with CS of 11.

### Perturbation analyses

We used the CMAP LINCS pilot phase dataset (GEO: GSE92742) for the analysis of perturbation effects. We used differential expression *Z* scores of the 978 landmark genes and 9,196 genes, which have been inferred with at least 95% recall.^44^ For knockdown experiments in HT29 and PC3 cells, we used the consensus gene signatures computed from at least six different short hairpin RNA (shRNA) signatures using a weighted mean procedure.^44^^,^^45^ For HEK293T we used an unweighted mean of individual shRNA signatures, since most perturbagens are not tested with sufficiently many shRNA fragments to obtain a consensus signature. For each perturbagen, we compare the differential expression *Z* scores of peripheral and core genes using t tests with FDR adjustment. We consider FDR < 0.05 as significant.

To ascertain the similarity between different sets of discriminative perturbagens across traits and cell types, we calculate the overlap coefficient between two sets *A* and *B* using the following formula:

$$\text{Overlap}\left(A,B\right)=\frac{\left|A⋂B\right|}{\min\left(\left|A\right|,\left|B\right|\right)}\text{.}$$

Since sets *A* and *B* are drawn from different total sets (or universal sets) *U*_*A*_ and *U*_*B*_ (the total set of perturbagens tested in two different cell types), we use a resampling approach to calculate an empirical *p* value for the significance of the overlap coefficient between two sets *A* and *B*. To obtain an empirical distribution of the overlap coefficient under the null hypothesis, we draw 1,000 random sets *A*_random_ with the same number of elements as *A* from the same universal set *U*_*A*_ and another 1,000 random sets *B*_random_ with the same number of elements as *B* from the same universal set *U*_*B*_ and calculate their overlap coefficients. We then obtain the rank *r*_empiric_ of the real overlap index of *A* and *B* compared with the 1,000 randomly generated overlap coefficients. This empiric rank *r*_empiric_ is then transformed into an empiric two-sided *p* value using the following formulas:

$$r_{\text{empiric}}^{\prime }=\frac{r_{\text{empiric}}}{1001}\text{,}$$

$$p_{\text{empiric}}=\min\left(r_{\text{empiric}}^{\prime },1-r_{\text{empiric}}^{\prime }\right)\times 2\text{.}$$

The final empiric *p* value is then adjusted using FDR over all combinations of traits and cell types.

### Transcription start site and enhancer analyses

We obtained the number of transcription start sites (TSSs) and enhancers per gene from Mostafavi et al.^2^ Originally, they were obtained by analyzing promoter regions identified by the FANTOM consortium.^46^ The TSS count, the number of hg19 CAGE peaks, was computed from FANTOM5 phase 1 and phase 2 data linked with each gene. Genes not present in the data were assigned the value 0. Active enhancer count was originally obtained based on the correlation of chromatin marks with gene expression.^47^ First, the union of all enhancer intervals per gene in a given biosample was compiled. The final count is the number of biosamples in which the gene is linked to at least one enhancer interval.

### Co-perturbation simulations

We simulated co-perturbation using GEARS.^48^ GEARS is a graph-based machine-learning framework designed to predict the effects of genetic perturbations on gene expression. It represents genes and perturbations as embeddings, leveraging gene co-expression and pathway similarity graphs to encode relationships. Using a graph neural network (GNN)-based encoder, GEARS integrates perturbation embeddings with gene embeddings and applies a cross-gene decoder to model downstream expression changes. The model accounts for combinatorial perturbations using a compositional module and improves accuracy with an autofocus direction-aware loss that prioritizes differentially expressed genes. By incorporating gene-gene interactions and systematic perturbation modeling, GEARS enables robust predictions of gene-expression changes in unseen perturbations. GEARS categorizes interactions based on interaction classes defined in Norman et al.^49^ using a regressor trained on the individual perturbation vectors (*A* and *B*), trying to predict the co-perturbation vector (*AB*):

$$c_{1}A+c_{2}B=AB\text{.}$$

It then calculates a magnitude value based on the length of the learned parameter vectors:

$$\text{Magnitude}={\left(c_{1}^{2}+c_{2}^{2}\right)}^{0.5}\text{.}$$

A low magnitude means that the co-perturbation expression is smaller than the sum of the individual perturbations, which indicates suppression. Conversely, a high magnitude means synergy. GEARS further classifies interactions as neomorphisms based on the goodness of fit of the regressor:

$$\;\text{Model}\;\text{fit}=\text{corr}\left(c_{1}A+c_{2}B,AB\right)\text{.}$$

A low model fit indicates that the co-perturbation expression vector cannot be linearly approximated by the sum of the individual vectors, indicating neomorphism.

We trained a model using the default settings with the co-perturbation dataset from Norman et al.^49^ For inference, we query the trained model for gene pairs which are either sourced from core genes, or peripherals with either weak or strong GWAS signal (HSPs), and sort the resulting model parameters in categories based on the query gene pair. The individual group count distribution of the top 100 and top 500 suppression and neomorphism values is then compared to the global distribution using a $\chi^{2}$ goodness-of-fit test.

## Results

### Separating core genes and peripheral genes

To explore the omnigenic architecture for UC, we aimed to identify a set of confident core genes for this disease. We gathered 379 genes from OMIM in which variants have been reported to cause Mendelian disorders matching established standardized clinical phenotype terms for UC^12^ and used them as core genes^11^ for model training (Table S2). Speos predicts novel core genes by integration of the network context of all genes with tissue-specific gene expression and GWAS signal using a GNN model.^13^ We constructed a multi-modal graph using protein-protein interaction and gene-regulatory and metabolic networks. In this network, 4,103,739 modality-typed edges link 17,097 genes and proteins. The graph nodes represent both genes and their encoded proteins. Another 336 (1.9%) disconnected genes are included in the analyses. To train a Speos model, we used the OMIM-derived gene set, our multi-modal network, UC GWAS^50^ summary statistics aggregated on the gene level,^28^ and tissue-specific gene expression in 72 tissues and cell types.^29^^,^^30^ For UC, Speos predicts 1,316 core-gene candidates with varying degree of confidence represented by a CS, with a higher CS representing higher model confidence (Figure 1B and Table S3). Similar to the OMIM-derived genes, the identified candidates in all CS bins are strongly enriched for phenotypically matched mouse knockout genes, significantly more so than GWAS genes alone (Fisher’s exact test, FDR < 0.05; for exact *p* values see Table S4, Figure 1B, and Figure S1) or genes prioritized by the recently proposed polygenic priority score (PoPs)^51^ (Figure S1B). In contrast to GWAS genes, OMIM-derived genes and Speos candidates are also significantly enriched for genes that are differentially expressed in individuals with UC^52^ (Fisher’s exact test, FDR < 0.05; for exact *p* values see Table S5, Figure 1B, and Figure S1). Overall, genes with a higher CS exhibit a stronger enrichment, as expected for high-confidence core-gene candidates, highlighting the reliability of our approach. These validations support the hypothesis that combining GWAS with gene-expression data and molecular networks improves the identification of genes with core-gene properties.^7^^,^^8^

In the omnigenic model, “core” and “peripheral” genes are not mutually exclusive binary distinctions, but instead there is a continuous increase of core-gene quality among refined groups of genes,^7^^,^^8^ which may be reflected in the Speos CSs. To increase contrast between core and peripheral gene sets for subsequent analysis, we consider as “UC core” the OMIM-derived training genes and the most confident predictions (CS 11) resulting in 693 confident core genes for UC. We further identified 16,117 genes with a CS of 0 as confident peripheral genes. Expectedly, the core genes show a stronger GWAS signal and selective constraint on average than peripheral genes (Figures 1C and S1; Table S6), indicating the biological validity of our core-gene set. The enrichment of the GWAS signal in core genes is still significant when controlling for LD, genetic conservation, and other co-variates (Figure S1E). However, we also noted several peripheral genes with strong associations to UC in GWASs (Figure 1D). This raises the question of how these HSPs differ from core genes. We started to address this question by dissecting salient patterns on the level of individual genes.

### Gene-level properties

#### Core genes

**Figure 2:**
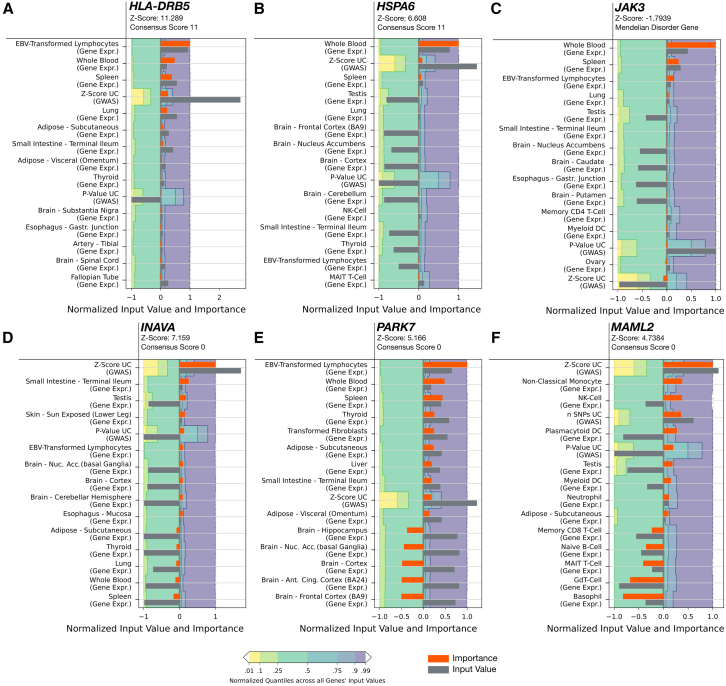
Feature importance scores for selected genes (A–C) Feature importance scores (orange bars) and input values for each feature (gray bars) of core genes. Positive importance scores indicate arguments in favor of core-gene prediction, while negative scores speak against core-gene prediction. Shown are the ten features with the highest score and five features with the lowest score. (D–F) Feature importance scores (orange bars) and input values for each feature (gray bars) of highly significant peripherals (HSPs).

The omnigenic model postulates that beyond the GWAS signal, expression in trait-relevant tissues especially is a fundamental characteristic of core genes.^8^ Even though the Speos-identified core genes show a stronger GWAS signal on average, many peripheral genes exhibit similar association strengths (Figures 1C and 1D). We therefore aimed to identify features that enable the model to distinguish core genes from peripheral genes. We selected core genes with diverse GWAS signal strengths and applied integrated gradients^37^ on the input features. “Integrated gradients” is an interpretable machine-learning technique that explains model predictions by attributing importance to input features. It compares the model’s response to an actual input against a baseline (a vector of zeros) and gradually transitioning between them while computing gradients at each step.^37^ The accumulated gradients reveal which features most influenced the prediction.^37^ We ranked individual features by strong arguments for (positive importance) or against (negative importance) their prediction as core gene. Genes from the HLA region, which encode proteins of the major histocompatibility complex (MHC), exhibit a particularly strong GWAS signal^53^ and are important known factors in UC pathophysiology.^53^^,^^54^ Surprisingly, despite its very large GWAS *Z* score, expression is a stronger feature for *HLA-DRB5*’s prediction (Figure 2A). While it appears surprising that high expression specifically in Epstein-Barr virus (EBV)-transformed lymphocytes is considered an important factor by the model, EBV infections have been shown to exacerbate UC flares and influence disease development by altering T cell responses.^55^^,^^56^^,^^57^^,^^58^^,^^59^^,^^60^ Expectedly, other tissues related to the immune system (whole blood and spleen) similarly have a high positive importance. Overall, the tissues and cells that are most decisive for UC core-gene identification are more specific for the immune system than for the colon, congruent with the current clinical guidelines for the treatment of UC, which are almost exclusively focused on modulating the immune response.^61^^,^^62^^,^^63^ However, not least because of its manifestation in the colon, UC is considered an intestinal disease. Consistently, previous work has identified a subset of variant genes that confer susceptibility to UC, presumably by altering epithelial integrity, such as *CDH1* (E-cadherin) and *ECM1* (extracellular matrix protein 1),^17^ both among the OMIM-derived disease-associated genes for UC. We find that these genes’ most important feature is not expression in the sigmoid colon—the GTEx tissue most likely to be affected by UC—but instead expression in related tissues, such as the terminal ileum (Figure S2). Similar to the Speos-identified expression features, a recent transcriptome-wide association study (TWAS) identified tissues and cells of the immune system including spleen and EBV-transformed lymphocytes as most relevant for UC.^64^ Like Speos, this study identified the terminal ileum as the most important part of the digestive tract, which is unexpected, as UC manifests only in the colon and not in the ileum or other parts of the intestine.^64^ The fact that the ileum is identified by both studies as the relevant intestinal tissue could be due to the relative abundance of specific cell types, which facilitates the identification of relevant patterns in clinically unaffected tissues.^64^ At the same time, it is important to note that an aberrant immune response is increasingly recognized as a decisive feature of UC, while the colonic tissue itself likely plays a subordinate role.^17^ The Speos-identified expression features are consistent with this emerging view of UC pathology and the involved mechanisms. This is also illustrated by prediction of *HSPA6* as UC core gene, which is dominated by gene-expression patterns in whole blood and spleen (Figure 2B). Mechanistically, HSPA6 reduces inflammation in UC by stabilizing anti-apoptotic Bcl-XL,^65^^,^^66^ such that reduced *HSPA6* expression increases the risk of UC^65^^,^^67^ whereas increased expression has protective effects.^65^^,^^68^

The OMIM-derived disease-associated gene *JAK3*^69^^,^^70^ has a relatively low GWAS *Z* score. It functions in the JAK/STAT signaling pathway (KEGG: HSA04630) that mediates cytokine signaling in different cell types and plays a crucial role in inflammatory bowel disease signaling (KEGG: HSA05321^71^). Nonetheless, *JAK3* has not been associated with any disease in GWASs.^72^ However, rare mutations in *JAK3* have been reported to cause a clinical manifestation of severe immunodeficiency.^73^^,^^74^^,^^75^ Speos feature importance scores indicate that *JAK3*’s salient pattern is its restriction to immune cells and other cells of the hematopoietic lineage^76^ found in whole blood and spleen^77^ (Figure 2C). Our finding that certain expression patterns can outweigh a missing GWAS signal demonstrates the added value of multi-modal machine learning and the power of GWAS and gene-expression patterns in a network context for the identification of core genes. We further conclude that expression in disease-relevant tissues is a key feature for identification as core genes.

#### Highly significant peripheral genes

Among the 16,117 genes that are not predicted as core genes at any confidence score greater than zero, a few stand out due to their highly significant GWAS *Z* score. These HSPs show a different pattern regarding input importance in comparison with core genes (Figures 2D–2F). In contrast to the most confidently predicted core genes, for which the expression patterns are the decisive feature, the strongest argument in favor of a (not realized) core-gene classification are their GWAS *Z* scores. Consistent with their classification as peripheral genes, the HSPs lack specific mechanistic links to UC but do influence general immunity and inflammation-related processes. For example, consistent with a peripheral regulatory function, expression of *INAVA*, the activator of the innate immune response, enhances inflammatory interleukin-1β (IL-1β) signaling but is not essential for it.^78^ In contrast to bona fide core genes, *INAVA* is not expressed in immune cells,^79^ leading to low expression in immune-specific tissues such as whole blood and spleen (Figure 2D). Instead, *INAVA* is expressed in mucin-producing goblet cells,^79^ highlighting the indirect role of the intestinal epithelium in UC as intermediary between microbiota and the immune system. A second example, *PARK7* (Figure 2E), named after its role in Parkinson disease, is expressed in almost all human cells.^80^ While it is also investigated for its role in the gut-brain axis, its mechanism of action in both organs is likely the suppression of reactive oxygen species, a mechanism involved in many diverse conditions.^81^ Lastly, *MAML2* (Figure 2F) is part of the mastermind-like family of genes, expressed in almost all tissues and a co-activator of the NOTCH pathway. This pathway governs an array of vital developmental processes.^82^ It is assumed that its role in UC is the regeneration of intestinal mucosal cells by regulating the differentiation of mucosal stem cells.^83^ However, as with *INAVA*, *MAML2* is an accessory regulator that is not essential for these processes. These patterns for exemplary core genes and HSPs are at the same time consistent with the current view of UC pathology^17^ and with central assumptions of the omnigenic model.^8^^,^^9^ We wondered whether these patterns extend beyond individual examples and may indeed be evidence for expression being a defining feature of the Speos-identified core genes.

**Figure 3:**
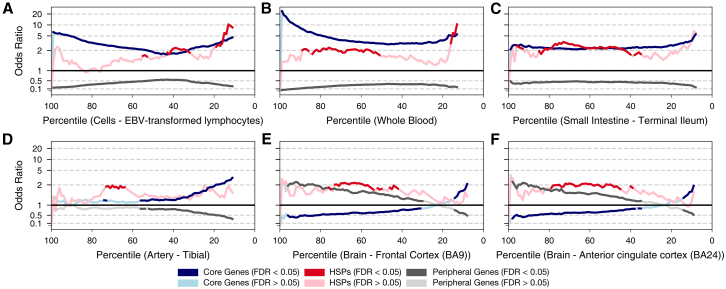
Enrichment of core genes, HSPs, and peripherals in selected tissues (A–F) Odds ratios of core genes (blue), HSPs (red), and peripherals (gray) in or above a given percentile of the genome after sorting genes according to their expression in the respective tissue or cell type (*x* axis). Darker colors indicate significant enrichment/depletion (Fisher’s exact test, FDR < 0.05; for individual *p* values see Table S7). HSPs are selected based on a GWAS *Z* score larger than 5 calculated from the UC GWAS GCST003045.

We therefore investigated genome-wide gene-expression patterns for the tissues that we and others^64^ previously identified as disease relevant (Figures 2A–2F and S3). We ranked genes by their expression in each tissue and calculated odds ratios of core genes, peripheral genes, and HSPs being among the most strongly expressed genes. Core genes are strongly enriched among highly expressed genes in disease-relevant tissues (Figures 3A–3C, Fisher’s exact test, FDR < 0.05; for exact *p* values see Table S7). In contrast, in tissues for which the example genes indicated a low assigned feature importance and that are unrelated to UC pathology (Figures 2A–2F), core genes are not over-represented (Figure 3D) or even depleted (Figures 3E and 3F), indicating that expression in these tissues is either unrelated or anti-correlated with the likelihood of being a UC core gene. HSPs, on the other hand, are lacking diseased-tissue specificity and instead show a similar expression pattern across all investigated tissues (Figures 3D–3F). Thus, as postulated by the omnigenic model, tissue-enriched gene-expression patterns are a key feature of UC core genes. This opens the questions of whether core genes can inform on central pathways underlying disease pathology and which mechanisms link genetic variation at HSPs to UC.

### Functional analysis

**Figure 4:**
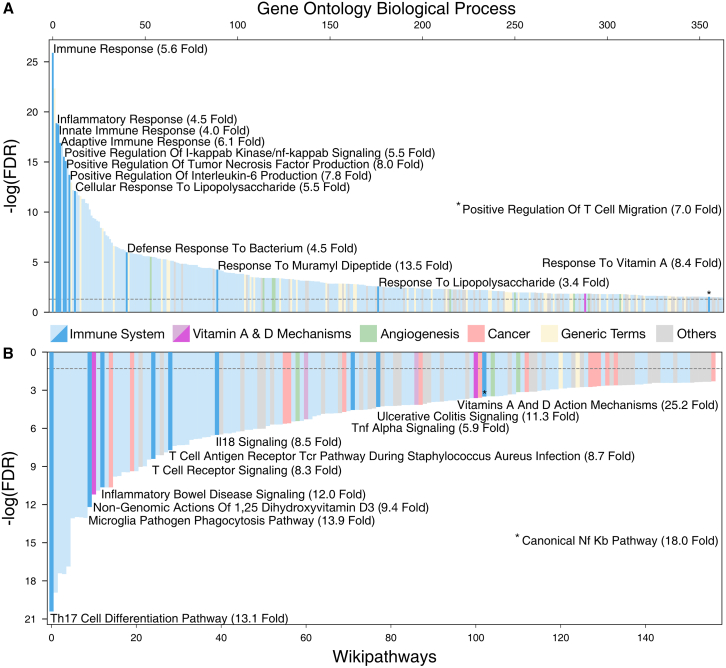
Gene set enrichment analysis Enrichment of Gene Ontology biological processes (A) and WikiPathway pathways (B) among UC core genes. Terms are colored by organizational group (see legend). Selected terms are plotted in darker shades and annotated with name and odds ratio. The dashed line represents the FDR cutoff at 0.05. Precise *p* values and the full list of significant terms can be found in Table S8. Pathways are plotted in blue and annotated with name and odds ratio. Precise *p* values and pathways plotted in gray can be found in Table S8.

Since core genes are expected to influence cellular functions that are immediately causal to the disease phenotype, we first explored which known pathways and functions are enriched among the confident core genes. Overall, two-thirds of significantly associated terms are related to the immune system or inflammatory responses (Figure 4 and Table S8). More specifically, we find an enrichment of pro-inflammatory signaling cascades, responses to bacterial surface proteins, and upregulation of T cell migration (Figure 4A and Table S8), all key aspects of UC.^16^^,^^17^^,^^18^^,^^19^^,^^20^^,^^21^^,^^22^^,^^23^ Our core genes further reflect the importance of vitamins A and D in UC,^84^^,^^85^^,^^86^^,^^87^ which may act as immunomodulators and tolerogenics,^85^^,^^87^ i.e., increasing the host’s tolerance to microbial stimuli. Finally, upregulation of angiogenesis and vascularization in order to heal mucosal lesions is frequently observed in UC^88^^,^^89^ (Figure 4A and Table S8). We find a similar picture among enriched pathways, bolstered by differentiation of T-helper 17 cells, a crucial cell type in UC,^90^ and respective disease signaling pathways (Figure 4B and Table S8). While the simultaneous involvement of inflammatory and angiogenic pathways leads to enrichments in certain cancer pathways, most enriched terms are centered around the physiologic aspects of UC. We therefore conclude that our confident core-gene predictions are representative for processes central to UC, more so than genes with less confident predictions (Table S8).

### Network-level properties

Naturally, pathway annotations are conceptual simplifications. Beyond annotated pathways, core-gene regulation and protein functionality are embedded in networks of regulatory interplay on the biochemical, transcriptional, and metabolic level. Thus, investigating which parts of the network are important for their prediction as core genes may lead to hypotheses about relevant causal mechanisms. We investigated the trait-specific importance of individual edges in our multi-modal network by applying integrated gradients^37^ to the message passed from node to node in the GNN’s underlying message-passing framework. Each model of the Speos ensemble assigns an importance score to every edge in the network, which cumulatively give rise to network-based core-gene predictions. To identify the most important edges, the absolute values of importance scores are scaled to [0; 1] and averaged across the ensemble. This procedure is repeated for every core gene, reflecting the relative importance of every edge for the individual core-gene predictions. Finally, each edge is attributed with the maximum score across all core genes, resulting in a trait-centered importance network.

**Figure 5:**
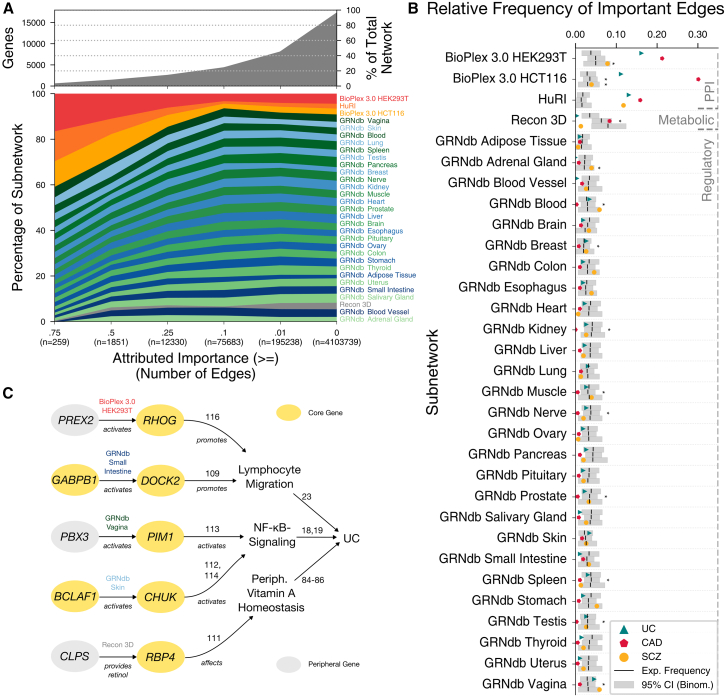
Edges with high importance scores (A) Distribution of edge types among edges above certain thresholds of attributed importance. Edge types are sorted by their distribution at ≥0.75 attributed importance. The gray graph above shows how many genes are still part of the network when the thresholds are applied. (B) Relative frequency of important edges (attributed importance ≥0.75) for core genes of UC, CAD, and SCZ. Black bars denote the relative frequency of edges leading toward core genes in their 1-hop neighborhood. Gray bars denote the 95% confidence interval (CI) around the expected relative frequency assuming a binomial distribution. Asterisks denote significant heterogeneity between traits in the importance of the respective subnetwork (binomial test, FDR < 0.05; for individual *p* values see Table S9). (C) Selected edges with attributed importance of 0.75 or higher for UC. Shown are the incident nodes and how the receiver nodes can influence the phenotype by affecting important processes. Edges between genes are labeled by type, and other links are annotated with the respective reference.

Intriguingly, the most important edges (>0.75 relative importance) are predominantly protein-protein interactions and regulatory interactions from disease-relevant tissues such as blood and spleen (Figure 5A). High frequencies of important regulatory connections in other tissues, such as skin, vagina, and pancreas, are an indication that the disease-causing mechanisms of UC have parallels in other tissues, potentially underlying clinically relevant extraintestinal manifestations.^91^^,^^92^^,^^93^^,^^94^^,^^95^^,^^96^^,^^97^

We aimed to identify which network modalities are most relevant for UC. However, to assess whether such modalities differ between different types of diseases, we also analyzed two additional traits with different molecular etiology: coronary artery disease (CAD) and schizophrenia (SCZ) (Note S1, Figure S4, and Tables S1, S3, and S4). CAD is a disease of the vessels supplying blood to the heart muscle and is the most common heart disease. SCZ is a psychiatric disorder characterized by severe psychotic behavior including hallucinations and disorganized patterns of thinking and speech. For all three diseases, protein-protein interactions are frequently assigned high importance scores, indicating the central role of this network modality for diverse traits (Figures 5B and S5; Tables S9 and S10). Physical interactions attributed with a high importance score for CAD include the physical binding of kallikrein-related peptidase 10 (*KLK10*) to apolipoproteins E and C1 (*APOE* and *APOC1*). Apolipoproteins play a vital role in the formation of lipoproteins, which transport lipids in the bloodstream.^98^ They are also involved in depositing lipids in arterial lesions, leading to atherosclerotic plaque buildup and, finally, CAD.^98^^,^^99^ *KLK10* is a tissue serine protease^100^ able to break down lipoproteins and apolipoproteins,^101^ inhibiting endothelial inflammation and atherosclerotic plaque formation in mice.^102^ Another striking pattern is the over-representation of regulatory edges with high importance scores for SCZ in tissues with regular microbial contact, such as colon, esophagus, stomach, and vagina (Figure 5B). Recent findings suggested a possible link between microbiome dysbiosis of these organs and the pathogenesis of SCZ.^103^^,^^104^^,^^105^

Compared to UC, important edges in CAD show an over-representation of metabolic connections (Figure 5B), including the transfer of glycogen, lactate, and creatine (Table S11). These metabolites play vital roles in CAD through glucose disposal and glycemic variability.^106^^,^^107^ Compared to UC, important edges in SCZ show an over-representation of regulatory connections in the adrenal gland, which has been suggested to be implicated in the pathophysiology of SCZ^108^ (Figure 5B). Thus, edges with high importance scores hold the potential to identify network modalities and, together with node importance features, the tissues that are important for the respective disease.

Protein-protein interactions play a vital role in all three traits and aid the identification of disease-modifying mechanisms with possible implications for drug development. Intriguingly, most high-importance edges in PPI networks connect proteins whose interplay has not yet been studied, opening numerous opportunities for subsequent interrogation (Table S9).

Since genetic variation at peripheral genes is assumed to account for the majority of heritability,^6^ we aimed to leverage our trait-specific subnetwork to explore how peripheral genes could exert their postulated influence on core genes. We selected regulatory edges incident to core genes with an attributed importance of 0.75 or higher to identify confident peripheral candidates for their direct influence on core genes or their encoded protein products (Figure 5C). These are examples of peripheral genes that our model interpretation linked to core genes that directly influence processes^84^^,^^85^^,^^109^^,^^110^^,^^111^^,^^112^^,^^113^^,^^114^ central to UC.^18^^,^^19^^,^^23^^,^^84^^,^^85^^,^^86^ These can be illustrated, e.g., by the physical association between the rac guanine nucleotide exchange factor *PREX2*, which activates the small G-protein cytoskeletal regulator *RHOG*,^115^ and the regulatory link between the transcription factor *GABPB1* that induces *DOCK2* expression,^27^ another cytoskeleton regulator, both of which are known to increase migration of lymphocytes.^109^^,^^116^ Similarly, *PBX3* and *BCLAF1*, respectively, regulate the expression of *PIM1* and *CHUK*,^27^ which are known to influence or even be part of the NF-κB signaling pathway.^112^^,^^113^^,^^114^ Finally, *CLPS* provides retinol to *RBP4*,^26^ which in turn is crucial for peripheral vitamin A homeostasis.^111^ Unsurprisingly, we found that the relationships are not exclusively from peripheral genes to core genes to the phenotype but often also run between core genes, a regulatory relationship not prominently considered in the omnigenic model.^8^^,^^9^

Thus, network connections in the local neighborhood of core genes that Speos weighs high reflect known disease mechanisms and point to promising avenues of future research. Next, we explored whether system-wide perturbation effects can illuminate direct and indirect functional connections within the network.

### Perturbation effects

Besides affecting core-gene function through physical interactions, peripheral genes are expected to influence traits by affecting core-gene expression through the respective cellular regulatory network.^8^^,^^9^ We explored whether specific peripheral genes influence core-gene expression and whether HSPs have a different regulatory impact on core genes than peripheral genes without a significant GWAS signal. As candidate HSPs, we considered genes that are less than 10 kb up- or downstream of GWAS SNPs with an individual *p* value cutoff of $5\times 10^{-8}$, which allows unambiguous identification of the source of the GWAS signal. To avoid genetic correlates, we discarded SNPs that reside less than 10 kb up- or downstream of any gene with Speos CS greater than 0 or in the same LD block as OMIM-derived genes or core genes with CS of 11. Using this stringent approach, we identified 17 confident independent HSPs for UC (Table S12) from two large-scale GWASs of predominantly European heritage (Table S12).

We wondered whether HSPs exert a large influence on traits because they occupy a unique position in the network, e.g., proximal to a larger number of core genes. We addressed this question using label propagation from UC HSPs through the network to inspect the resulting distribution of core and peripheral genes. HSPs are significantly closer to core genes than to peripheral genes (Note S2, Figure S6, and Table S13). However, this was not specific to HSPs but was observed also when using other peripheral genes as the starting point. This likely is a consequence of the position of UC core genes in the network, as they are more central than peripheral genes according to classical graph centrality measures such as betweenness (U test, *U* = 7,441,160, *p* = 1.22e−59), degree (U test, *U* = 7,901,004.5, *p* = 6.30e−89), and PageRank (U test, *U* = 6,858,784.5, *p* = 1.87e−30). Thus, core-gene encoded proteins have a central position in the network, but proximity to these does not distinguish HSP proteins from those encoded by other peripheral genes.

**Figure 6:**
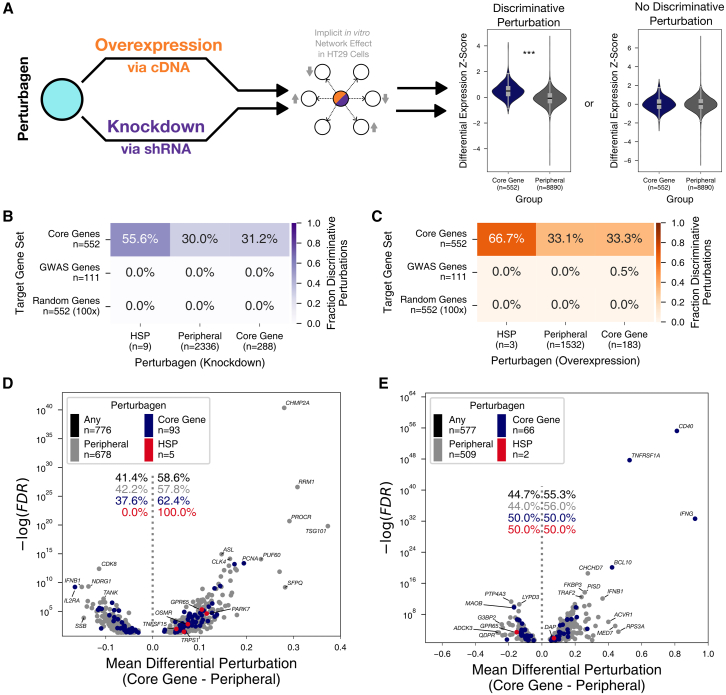
Differential perturbation analyses (A) Overview of differential perturbation analysis. For each knockdown and overexpression gene, the resulting change in expression in HT29 cells is obtained from cMap in the form of *Z* scores. If the core genes of a trait have significantly different *Z* scores than peripherals, we score the perturbagen that is responsible for this perturbation as a significant perturbagen. (B) Fraction of significant knockdown perturbagens for UC core genes in HT29 cells (upper row) contrasted with the fraction of significant perturbagens for GWAS genes (middle row) and randomly selected genes (lower row, 100 repetitions) (t test, FDR < 0.05; for individual *p* values see Table S14). (C) Fraction of significant overexpression perturbagens for UC core genes in HT29 cells (upper row) contrasted with the fraction of significant perturbagens for randomly selected genes (lower row, 100 repetitions) (t test, FDR < 0.05; for individual *p* values see Table S15). (D) Volcano plot of individual significant knockdown perturbagens. Shown is the resulting mean in differential perturbation of core genes versus peripherals and the accompanying FDR (t test). (E) Volcano plot of individual significant overexpression perturbagens. Shown is the resulting mean in differential perturbation of core genes versus peripherals and the accompanying FDR (t test).

We wondered whether the central position of core genes in the network topology is also evident in the dynamic functional response of the system to perturbations. Systematic perturbation datasets map the effects of perturbing one gene on expression changes of other genes without requiring an explicit model of the underlying network. We used the Library of Integrated Cellular Signatures (LINCS) provided by the connectivity map initiative (GEO: GSE92742), which details expression differences resulting from knockdown or overexpression of hundreds to several thousand genes across 17 diverse cell lines.^44^ The transcriptional responses to these perturbations are measured using 978 landmark genes from which the expression of 9,196 additional genes is inferred with a recall of at least 95%,^44^ where recall is defined as the proportion of less similar profiles in a simulated reference distribution serving as null hypothesis. We first explored perturbations in HT29 cells, a model cell line for UC.^117^ Each knockdown perturbation is carried out with multiple shRNAs, and the measured transcriptional changes are aggregated to one consensus signature per perturbagen.^44^^,^^45^ We considered a perturbagen to be “discriminative” if the distribution of expression changes of our UC core genes differs significantly from that of peripheral genes (Figure 6A), i.e., if core genes are significantly more up- or downregulated than the peripheral genes, or vice versa.

In response to knockdown perturbations, about every third perturbagen leads to a discriminative change of UC core-gene expression, irrespective of whether core or peripheral genes are perturbed (two-sided t test, FDR < 0.05; for individual *p* values see Table S14 and Figure 6B). In contrast, when we assessed discriminative responses of randomly selected gene sets, essentially no significant discrimination was observed. Surprisingly, similar to the randomly selected genes, GWAS-identified genes showed essentially no differential effect compared to non-GWAS genes when either core genes or peripheral genes were selected as perturbagens, indicating that the contrast in response to perturbation elsewhere in the genome is indeed a property of core genes. We wondered whether core genes are deregulated in a consistent manner by the different perturbagens, or if, while being deregulated as a set, they show overall patterns that are more specific to the individual perturbagens. We found that 86.7% of core-gene responses are uncorrelated, while only 0.2% are moderately to strongly correlated (Spearman’s rho <0.25 and >0.5, respectively) with others, indicating that the order of differential expression between core genes differs substantially from one perturbagen to another. Since the post-perturbation expression change vectors are denoised consensus signatures,^44^^,^^45^ we consider it unlikely that the low correlation of responses is due to sampling variation of individual responses but instead reflects actual differences in perturbation responses between core genes. Intriguingly, five out of nine HSPs among the knockdown perturbagens lead to significant discriminative perturbations of core genes but not of random or GWAS genes (two-sided t test, FDR < 0.05; for individual *p* values see Table S14 and Figure 6B). Even though this observation involves only nine HSPs, this is a significantly higher proportion of discriminative perturbagens compared to discriminative perturbations caused by peripheral genes and core genes (one-sided Z test, *Z* = 1.66, *p* = 0.048).

Overall, the same patterns emerge among the overexpression perturbation experiments. Almost every third gene is a significant discriminative perturbagen for UC core genes (two-sided t test, FDR <0.05; for individual *p* values see Table S15 and Figure 6C), irrespective if the perturbagens are sampled from core genes, peripherals or HSPs (one-sided Z test, *Z* = 1.23, *p* = 0.108). Again, when replacing the analyzed core genes with GWAS genes or randomly selected genes, only very few perturbagens lead to a discriminative perturbation of GWAS genes but not of random genes. Furthermore, discriminative perturbations leading to either concurrent up- or downregulation are equally common (Figures 6D and 6E). Thus, perturbation due to knockdown and overexpression frequently leads to patterns that discriminate between core and peripheral genes. However, the direction-of-perturbation effects on core genes do not necessarily have to be aligned in the same direction but could also affect parts of core-gene sets in opposite directions. This would result in an increase of variance compared to peripheral genes in response to perturbation. Indeed, a smaller proportion of perturbagens leads to a significant increase in variance of core-gene expression (one-sided F test, FDR < 0.05; for individual *p* values see Table S14; Figures S7A and S7B), a pattern that is also not observed for GWAS genes and randomly selected genes. However, most perturbagens lead to either a concordant mean shift or an increase in variance of UC core genes but not both, indicating that the mechanisms underlying these responses might have different biological implications (Figures S7C and S7D). These findings indicate that the identified core genes have a central position in cellular regulatory networks that is different from randomly selected genes and, importantly, also from GWAS genes. This finding not only strengthens the validity of the identified core genes but also provides strong support for a central assumption of the omnigenic model, namely that regulatory effects of many genetic variants converge on a small set of core genes.

**Figure 7:**
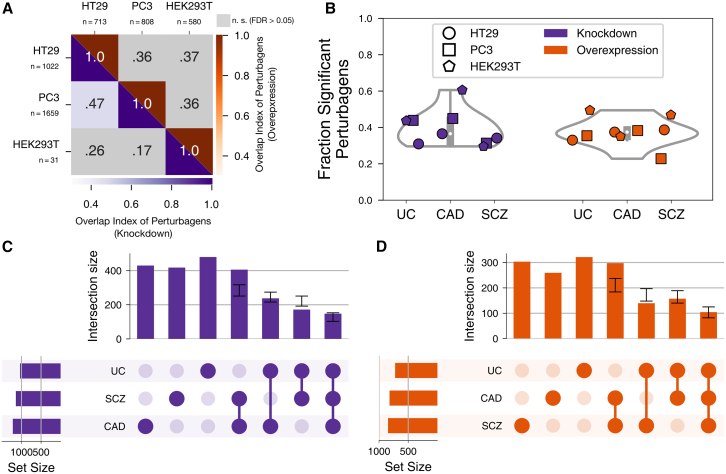
Significant discriminative perturbagens of UC, CAD, and SCZ (A) Overlap indices of significant perturbagens for UC across three cell types. Significant overlap indices are shown in color (FDR < 0.05; for individual *p* values see Table S16) and otherwise in gray. (B) Fraction of significant perturbagens across three traits and three cell types. The resulting total distribution for each perturbation type is shown as a violin plot in the background. (C and D) UpSet plots of significant knockdown (C) and overexpression (D) perturbagen sets for three traits in HT29 cells. Black intervals above intersection bars indicate the 99% confidence interval (CI) of an empiric null distribution obtained by 1,000 random draws. Bars ending within CIs indicate that the respective sets are independent of each other, while bars ending before reaching the CI indicate significant depletion.

An important question is whether the observed perturbation patterns are specific, both regarding the set of core genes for UC and regarding the cells in which the perturbations are assessed. To address the latter, we complemented our analyses using data for pancreatic cancer (PC3) and human embryonic kidney (HEK293T) cell lines. We observed a similar rate of significant discriminative perturbations in these two cell lines, indicating that the central position of UC core genes in the regulatory network is independent of cell identity (Figure S8 and Table S14). Intriguingly, discriminative perturbagens do not overlap significantly across cell types (Figure 7A and Table S16). This indicates that the underlying network transferring the perturbation effects to core genes differs between cell types.

Wondering whether the robust concerted effect of genetic perturbations might be restricted to UC core genes, we repeated the same analyses using the previously established core genes of CAD and SCZ, two traits highly dissimilar to UC. Intriguingly, a similar proportion of discriminative perturbations emerges for these orthogonal core-gene sets (Figures 7B, S9, and S10; Table S14), indicating that concerted perturbation is a general property of core genes and their position within regulatory networks. Only for SCZ do GWAS genes show a weaker albeit similar pattern, which might be indicative of their shared functional and spatial localization in the synapse.^118^ This indicates that for some traits, a GWAS is better suited to detect genes with core-gene properties and that prioritization tools such as Speos likely benefit from gene-expression data with a higher spatial and/or cellular resolution of complex organs like the brain when applied to such traits. However, while the proportion of discriminative perturbagens is nearly identical across traits, the majority of discriminative perturbagens is trait specific (Figures 7C and 7D). The observation that core-gene sets for such diverse traits are coherently deregulated upon overexpression or knockdown of genes elsewhere in the genome suggests that it is a general property of core genes that these are at the center of the regulatory network. Consistently, 68% of the 3,302 knockdown genes in HT29 cells and 72% of the 2,160 overexpressed genes discriminate core genes from peripheral genes of at least one trait. Thus, the regulatory network mediating these effects is wired in a way that a large fraction of the genome exerts non-zero effects on phenotypically important genes (see mouse validation data in Figure 1B) via coordinated modulation of core-gene expression. Lastly, we wondered whether the set of discriminative perturbagens is more similar among related diseases. To this end, we included rheumatoid arthritis (RA), an autoimmune disease of the joints that shares genetic and pathophysiologic features with UC,^12^ and Alzheimer disease (AD), which has symptomatic^119^ and pathophysiologic^120^ similarity with SCZ, in our comparisons (Note S3, Figure S11, and Tables S1, S3, and S4). Indeed, related traits share more discriminative perturbagens than unrelated traits in all three tested cell types (Figures S12–S15 and Table S16), indicating that the underlying omnigenic architectures are similar for related diseases. The observation of significant discriminative perturbation of core genes for five different traits bolsters the conclusion that core genes are at the center of an intense regulatory exchange. Indeed, our core genes have an increased number of TSSs and active enhancer elements,^2^ indicating a higher regulatory complexity in comparison with peripheral genes (Figures S12D and S12E) and providing additional support and a biochemical rationale for the observed results.

Given the interconnected complexity of biological systems and the large number of discriminative perturbagens, we wondered whether the simultaneous perturbation of two or more core genes might lead to non-linear effects. Co-perturbation analyses compare the impact of perturbing a pair of genes individually and in combination and are therefore uniquely poised to detect genetic interactions underlying the formation of emergent phenomena. These genetic interactions lead to genome-wide effects that differ substantially from the sum of the individual perturbations. Especially important are suppression effects, when the joint perturbation of two genes leads to attenuation of the individual effects, and neomorphism effects, when the joint perturbation effect differs substantially from the individual effects.

**Figure 8:**
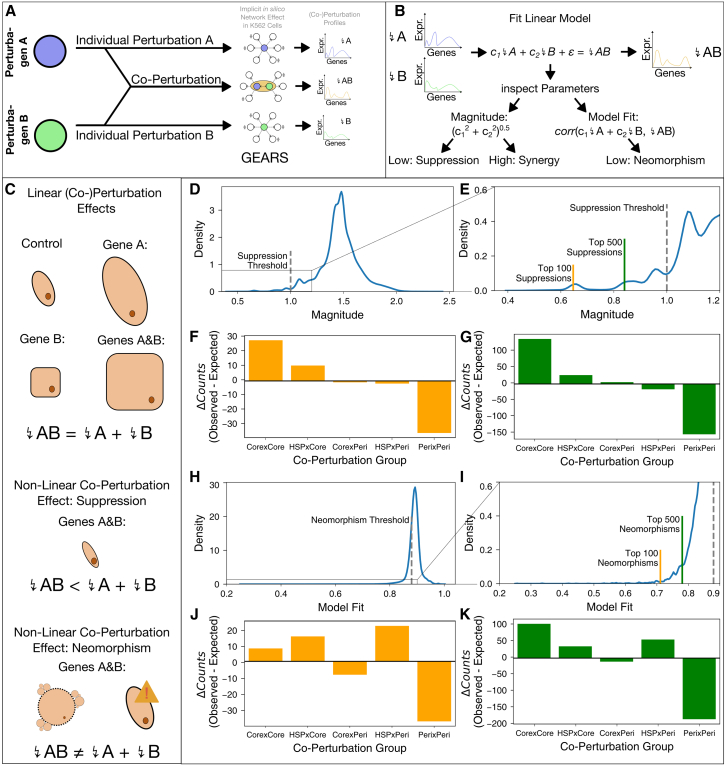
Co-perturbation simulations (A) GEARS is trained both on single and co-perturbations. When selecting a pair of genes, it predicts individual perturbation effects and co-perturbation effect. (B) Following the predictions of individual and co-perturbation effects, GEARS fits a linear model that aims to predict the co-perturbation effect from the individual perturbation effects. Depending on the magnitude of model parameters and goodness of fit, suppression, synergy, and neomorphism effects can be obtained. (C) Visual examples of linear additivity and non-linear suppression and neomorphism effects. (D and E) Magnitude of model parameters of 150,000 simulated co-perturbation pairs. High values indicate synergies, while low values indication suppression effects. (F and G) Difference in observed versus expected co-perturbation pairs among the top 100 (F) and top 500 (G) suppression effects, grouped by gene class. (H and I) Model fit of 150,000 simulated co-perturbation pairs. Values close to 1 indicate linear additivity, while low values indicate that co-perturbation effects are distinct from the individual perturbation effects, i.e., neomorphisms. (J and K) Difference in observed versus expected co-perturbation pairs among the top 100 (J) and top 500 (K) neomorphism effects, grouped by gene class.

While it is both laborious and expensive to comprehensively measure second-order co-perturbations, recently deep-learning models have been developed to simulate the effects of co-perturbations based on a limited training set.^48^^,^^49^^,^^121^ GEARS is a graph-based machine-learning framework designed to predict the effects of genetic perturbations on gene expression both for individual perturbations and co-perturbations (Figure 8A). By subsequently fitting a regression model on the simulated post-perturbation expression vectors, the GEARS^48^ framework further classifies genetic interactions into subclasses previously defined by Norman et al.^49^ The magnitude of the interaction is obtained by evaluating the length of the learned parameter vectors of individual and co-perturbations, where low values indicate suppression and large values indicate synergy (see methods and Figure 8B). Low goodness of fit of the regressor, however, means that the co-perturbation vector cannot be adequately approximated as a linear combination of the individual perturbation vectors and is therefore considerably neomorphic (see methods and Figure 8B). We trained a GEARS^48^ model (Figures 8A and 8B) on an experimentally established set of individual and joint overexpression of 115 genes^49^ and evaluated the predicted effects of 117,568 co-perturbations by 9,109 genes (Table S17). We considered co-perturbation pairs according to our classification of individual genes into UC core genes, peripheral genes, and HSPs and compared the frequency of the group combinations among the strongest neomorphism and suppression effects (Figure 8C). Interestingly, co-perturbations of UC core genes lead to strong suppression and neomorphism effects more often than expected by chance (Figures 8D–8K, χ^2^ test, *p* < 0.05; for individual *p* values see Table S18). One co-perturbation of core genes that is predicted to lead to a weaker effect than the sum of its individual perturbations is the gene pair *NFKB1*-*MAP2K3* (magnitude 0.6). These genes are both involved in “cellular response to lipopolysaccharide” (GO: 0071222) and are respectively crucial for NF-κB and p38 MAPK signaling, two pathways that are known to be highly similar in terms of both their activators and their resulting effects on the transcriptome.^122^ Thus, overexpression of either of the genes might partially saturate the pro-inflammatory signal, reducing the added effect of the joint perturbation. In contrast, the gene pair *BAX*-*NFKBIA* is predicted to lead to strong neomorphic synergy (magnitude 2.1, model fit 0.65). While both genes are involved in the apoptosis pathway,^43^ *BAX* encodes the pro-apoptotic protein Bax^123^ whereas *NFKBIA* inhibits the NF-κB pathway, which has been shown to induce apoptosis if inhibited.^124^ In contrast to the previous example, apoptosis is a bistable process that is activated by reaching a certain threshold.^125^ Thus, joint overexpression of both genes might have a potentiating effect, triggering apoptosis and thereby greatly altering the cell’s transcriptome. Remarkably, co-perturbation simulations of core genes and HSPs, but not peripheral genes in general, also predict strong suppression and neomorphism effects. Thus, among the peripheral genes, HSPs stand out in that they are predicted to strongly interact with core genes in co-perturbations. However, given that only four of our HSPs are among the perturbagens available in GEARS, this initial finding warrants further examination once more comprehensive datasets and methods are available. Taken together, the results of our co-perturbation analyses provide a first insight on an important issue of the omnigenic model: Even if concerted regulatory effects on and the precise expression changes of core genes were known, inferring phenotypic change and the emergence of trait-specific symptoms remains non-trivial due to complex interdependencies between core genes. By showing that even second-order co-perturbations of UC core genes can either attenuate or potentiate each other, we highlight another layer of complexity, the understanding of which is crucial for the omnigenic model to fully realize its potential. Since these analyses are based on simulations, more comprehensive datasets are necessary to experimentally validate these predictions.

## Discussion

The omnigenic model is a theoretical framework aiming to explain the genetic architecture of complex traits, including diseases. While some supporting evidence for this model has been gathered by us^13^ and others,^126^^,^^127^^,^^128^^,^^129^^,^^130^ it is not entirely clear how generally this model can be applied and which, if any, of the specific assumptions and conclusions may need to be refined. A challenge to answering these questions is that the postulated core genes are unknown for most traits and that the mechanisms by which most peripheral genes and their encoded products influence core processes are therefore difficult to study. For further assessment of the omnigenic model and, hence, for the understanding of many complex traits, it is not only important to reliably identify core genes but also to understand which peripheral genes influence these through which molecular network paths. Furthermore, the role of tissue-specific expression, and thus the underlying question of why a given disease manifests in one tissue and not in others, are highly relevant in relation to the omnigenic model.

Focusing on the autoimmune disease UC, we found that core-gene predictions by our previously developed deep-learning framework Speos^13^ are strongly driven by high expression in spleen and blood and specifically in EBV-transformed lymphocytes. The absence of colon tissue was surprising; however, a recent TWAS similarly failed to produce evidence for the sigmoid colon as a relevant tissue and, moreover, positively identified the same tissues and even cell types as relevant for UC as we did.^64^ In fact, this is consistent with emerging views on UC, which emphasize the autoimmune nature of the disease.^16^^,^^17^^,^^61^^,^^62^^,^^63^ Accordingly, the fact that UC manifests in the colon is a consequence of the specific microbial fauna in this part of the digestive system to which a “diseased” immune system displays an overshooting response.^16^^,^^17^^,^^61^^,^^62^^,^^63^ Given this view on UC etiology, Speos reliably weighs expression in the disease-relevant tissues as decisive for core-gene identification.

One key observation from the broader analysis of relevant features is that the identified core genes exhibit a high specificity for the disease-relevant tissues. In contrast, HSPs lack this specificity and are more uniformly expressed across all tissues. Thus, in line with the omnigenic model, tissue-specific expression patterns are a gene-level feature that differentiates core and peripheral genes.

When exploring which network-level features, i.e., which links are most relevant for core-gene identification, protein-protein interactions stand out with a central role across traits. However, the interpretation of many of the highly weighed interactions is difficult because most have not yet been extensively studied outside of large-scale interactome studies, thereby providing opportunities for future research. The solitary important role of metabolic connections in CAD matches the clinical importance of nutrition and metabolism in CAD and thus demonstrates that disease-specific features are well captured by our analysis.

An important question for the understanding of biological systems and specific aspects of the omnigenic model regards the dynamic influence of peripheral and core genes on each other. We therefore investigated how core genes respond to perturbation by peripheral genes that are then conveyed to core genes through the underlying molecular network. We found that core genes are at the center of the molecular regulatory landscape, with more than two-thirds of the tested perturbagens significantly affecting the core-gene set of at least one trait differently than peripheral genes. These perturbations often lead to either concurrent up- or downregulation of core-gene sets, which is likely the result of co-regulatory programs among these functionally related genes.^131^^,^^132^ The aligned regulation of core genes provides strong experimental support for the central assumption of the omnigenic model, namely that the impact of peripheral gene perturbations converges on core genes.^8^ A smaller set of perturbagens increases the variance of core-gene expression characterized by simultaneous up- and downregulation of subsets of core genes, potentially reflecting antagonistic functional and regulatory relationships, as exemplified by *IL1* and *IL1RN*.^21^ Congruently, our data suggest that the distinguishable response to perturbation is a property of the set of core genes rather than of the individual perturbagen. In stark contrast to the high frequency of concerted perturbation for core genes, this effect was not observed for similarly sized random gene sets, and even for GWAS-identified disease-associated genes the proportion of discriminative perturbagens never exceeded 3%, except for SCZ, where the GWAS detects more genes in direct functional relationship with the trait’s physiology.^118^ Across traits, however, the ubiquity of concerted response to perturbations points to a uniqueness of the identified core genes within the organization of biological systems that may well be related to their role as core disease-associated genes. Furthermore, although we found that the proportion of discriminative perturbagens is comparable across cell lines, the actual perturbagens, and thus the network paths that link these to core genes, differ between cell types. Within the same cell type, however, core genes of related traits are affected by similar perturbagens, indicating that related traits share a similar omnigenic architecture within the same cell type. Understanding the central position of core genes in biological systems both conceptually and mechanistically will be an important question for future studies.

Technically, the ability to identify relevant tissues with Speos and the tissue specificity of omnigenic architectures together may help to make their identification via *trans*-eQTL (expression quantitative trait locus) mapping more effective.^7^ The disease-relevant tissues we identified can provide prior information on potentially informative tissues and genes and thereby drastically reduce the statistical burden placed upon *trans*-eQTL studies, enabling an independent confirmation of core-gene predictions.

Given the large number of discriminative perturbations, many genetic variants can be expected to occur concurrently, which makes it critical to explore potential synergistic and attenuating effects of co-perturbations, i.e., genetic interactions. Our results from co-perturbation simulations suggest that co-perturbations of UC core genes lead to genetic interactions significantly more often than expected by chance, whereby both suppression and neomorphism are enriched. The occurrence of genetic suppression is likely explained by the enrichment of core genes in disease-relevant processes (Figure 4).^133^ In a disease context, these will correspond to alternative functional variants in different disease-associated genes of the same pathway that will have a similar or identical impact. For less impactful variants that are more subtle than the overexpression perturbations tested herein, however, the effects might still be additive. Less readily explained and potentially more impactful for disease etiology are neomorphic interactions, in which the simultaneous alteration of the expression of several core genes is expected to have compounding effects linked to the emergence of phenotypic changes that would not be expected for individual core genes. Together with the surprisingly high rate of discriminative perturbations of core genes by peripheral genes, this opens the possibility that high-impact genetic variants in peripheral genes indeed have the potential to concertedly influence the expression of a combination of core genes that might exhibit genetic interactions and thereby could influence disease in unforeseen ways. Thus, given that substantial effort is being directed toward the discovery of core genes for various traits,^13^^,^^126^^,^^127^^,^^128^^,^^129^^,^^130^ our results indicate that future lines of inquiry will also have to focus on their complex joint effects on the trait.

Another key question is why some peripheral genes have such a drastically stronger effect size in GWASs than most other peripheral genes. We introduced the concept of HSPs to probe the existence of the postulated “peripheral master regulators,” which are expected to have two properties. First, they are expected to concertedly influence core-gene expression,^9^ a behavior that we have shown is ubiquitous across the genome. Second, for this peripheral gene to become a master regulator, the core genes it concertedly influences also need to have a shared direction of effect on the phenotype.^9^ This would allow the peripheral gene to have a sizable influence on the phenotype, making it detectable in GWASs.^9^ The observed abundance of concerted regulation indicates that the coordinated impact on core genes, i.e., meeting the first requirement, is more the rule than the exception. Although we observe an overall alignment of perturbation effects on core genes as a set, the individual response of each core gene differs between perturbagens. This suggests that virtually any peripheral gene has the potential to act as “peripheral master regulator” if the core genes it affects (most strongly) have a coordinated effect on the phenotype, i.e., if it also fulfills the second condition. However, given the enrichment of strong genetic interactions between core genes, it becomes evident that the effect on the phenotype by a concurrently deregulated set of core genes cannot easily be estimated as a sum of their individual effects, greatly complicating their interpretation. Albeit limited due to their scarcity, our results indicate that HSPs exhibit genetic interactions with core genes more often than expected, directly implicating them in the process. However, given that the set of HSPs for UC is so small, the alignment of concerted regulatory directions of effect between the peripheral genes and the core genes on the one hand and the directions of effects of the core genes on the phenotype on the other hand might be rare. This rarity indicates that the effects of concertedly regulated core-gene sets on a phenotype are commonly in different directions, so that often the concerted regulation does not cause a significant impact on disease occurrence. This simple additive model is further complicated by the effects of genetic interactions that we observe in the co-perturbation analysis. Alternatively, it has been argued that the “peripheral master regulators” are under strong selective constraint and might therefore not often be observed in GWASs,^9^^,^^134^^,^^135^ indicating that our HSPs are part of a GWAS-identifiable subset of all existing master regulators. Given that negative selection has a strong impact on shaping the genetics underlying complex traits,^134^ both options are plausible and not mutually exclusive. Thus, as we have shown that the first requirement of “peripheral master regulators” is often met and that establishing the second requirement is more complex than anticipated, future studies are expected to focus especially on the latter, investigating how complex interdependencies in core-gene sets give rise to consistent disease phenotypes. Existing efforts to systematically map genetic interactions in human cells will provide important data for this effort.^49^^,^^136^^,^^137^ It has been demonstrated by theoretical approaches^9^ that the concerted regulatory effect on core genes, which we demonstrated qualitatively (Figures 6B and 6C), could explain how a substantial fraction of the heritability of complex traits is spread across the genome. Since this requires the direction of effect of core-gene variants on the phenotype to be known,^9^ subsequent endeavors might extend our results by quantitatively describing the individual perturbagen’s contributions to heritability via modulation of core genes.

A key challenge in applying the omnigenic model is the identification of core genes. The difficulties arise on one hand from genes having different degrees of “core-ness.”^8^ More importantly, however, especially for complex traits, disease etiology often involves different molecular processes in different cell types that synergize toward a pathologic imbalance of homeostasis. Correspondingly, current approaches to define core genes for a trait vary from strict selections for well-understood and usually simpler traits,^6^ through literature-based curated gene lists,^129^^,^^130^ to unbiased integrative approaches often involving machine learning.^13^^,^^129^ As the latter require positive examples as training data, we leverage standardized clinical phenotype terms to query OMIM,^12^ recognizing the central role of Mendelian disorder genes in pathology^11^ and their overlap with common disease forms.^12^ Here, the use of multiple query terms provides broader coverage of the underlying diversity of molecular processes in different cell types that contribute to a complex trait.^12^ Evaluation of the resulting core-gene candidate sets is inherently challenging. However, we believe that the systematic evaluations in this and our previous study^13^ go a long way to not only demonstrate the relevance of the identified genes for the respective traits but also confirm several predicted properties of core genes and, thus, conceptually support the omnigenic model.

In conclusion, our results provide evidence for several central assumptions of the omnigenic model. First, we demonstrate that the expression of core genes as well as their regulation is centered around disease-relevant tissues and cell types. Further, we qualitatively demonstrate that concerted regulatory effects of peripheral genes on core genes are ubiquitous and that the networks connecting these peripheral genes to core genes have a strong cell-type specificity. Finally, we show that this ubiquitous concerted regulatory effect on core genes might frequently lead to genetic interactions, thereby allowing peripheral genes to influence disease in unforeseen ways. Future endeavors are expected to study how diseases arise from the individual effects and complex interplay between sets of core genes in order to complement our findings with quantitative estimates of effect sizes. This will enhance our understanding of the omnigenic architectures of complex diseases with far-reaching ramifications for diagnosis, prevention, and drug development.

## Data and code availability

All data supporting the findings described herein are available in the article and its supplemental information files. All datasets used in this study are already published and were obtained from public data repositories (see web resources). Data produced during this study, such as the raw results files, the trained model parameters, and large results files are available at https://zenodo.org/records/14035135. The code is open source, implemented in Python, and freely available. The code to reproduce the analyses mentioned herein is available at https://github.com/fratajcz/speos/tree/novel.

## Acknowledgments

This work was supported by the European Union’s Horizon 2020 Research and Innovation Program (project ID 101137201, CLARITY [P.F.-B.]) and the Free State of Bavaria’s AI for Therapy (AI4T) Initiative through the Institute of AI for Drug Discovery (10.13039/501100018833AID) (P.F.-B.). F.R. is supported by the Helmholtz Association under the joint research school Munich School for Data Science—MUDS. This work was supported by the BMBF-funded 10.13039/501100018929de.NBI Cloud within the German Network for Bioinformatics Infrastructure (031A532B, 031A533A, 031A533B, 031A534A, 031A535A, 031A537A, 031A537B, 031A537C, 031A537D, and 031A538A).

## Author contributions

Conceptualization, M.H. and P.F.-B.; implementation and analyses, F.R.; writing, F.R., M.H., and P.F.-B.

## Declaration of interests

The authors declare no competing interests.

## Web resources

BioPlex 3.0 edge lists, https://bioplex.hms.harvard.edu/interactions.php
Co-perturbation data, https://dataverse.harvard.edu/api/access/datafile/6154020
Co-perturbation simulation framework GEARS and GEARS library, https://github.com/snap-stanford/GEARS
Gene evolutionary rate profiling scores across 91 mammals, https://ftp.ensembl.org/pub/current_compara/conservation_scores/91_mammals.gerp_conservation_score/
GRNdb edge lists, http://grndb.com/download/
GWAS summary statistics, https://doi.org/10.5281/zenodo.3629742
Human protein-coding genes, https://www.genenames.org/download/statistics-and-files/
Human Gene Ontology annotations, https://current.geneontology.org/products/pages/downloads.html
HuRI edge list, http://www.interactome-atlas.org/download
LINCS perturbation data, https://www.ncbi.xyz/geo/browse/?view=series, GEO: GSE92742
LOEUF constraint metric, https://gnomad.broadinstitute.org/data#v4-constraint
Median gene expression in blood cells, https://v19.proteinatlas.org/download/rna_blood_cell.tsv.zip
Mouse knockout genes, http://www.informatics.jax.org/allele (selecting “Targeted” from “Generation Method” and “Null/knockout” from “Allele Attributes”)
PoPs gene prioritization scores, https://www.finucanelab.org/data
Recon3D, https://vmh.life/ (processed using ReconParser)
ReconParser, https://github.com/fratajcz/reconparser
Reference datasets of European ancestry for LD correction in MAGMA, https://cncr.nl/research/magma/
Speos, https://github.com/fratajcz/speos/
Tissue-specific median gene-expression values, https://storage.googleapis.com/gtex_analysis_v7/rna_seq_data/GTEx_Analysis_2016-01-15_v7_RNASeQCv1.1.8_gene_median_tpm.gct.gz
Transcription start sites and enhancer elements, https://zenodo.org/records/6618073
WikiPathways pathway annotations, https://zenodo.org/records/13933011

## Supplemental information

Document S1. Figures S1–S15, Table S12, and Notes S1–S3

Table S1. Number of included genes, OMIM-derived genes, final core genes, and further summary statistics

Table S2. Standardized clinical phenotype terms

Table S3. Results from Speos runs and gene-level information

Table S4. Enrichment of mouse knockout genes among Speos candidate/Mendelian/GWAS genes

Table S5. Enrichment of differentially expressed genes among Speos candidate/Mendelian/GWAS genes

Table S6. Results of Tukey’s HSD tests comparing the loss of function observed/expected upper bound fraction (LOEUF) between predicted core genes, GWAS genes, and peripheral genes

Table S7. Fisher’s exact tests for tissue-enriched expression patterns of core genes, peripherals, and HSPs

Table S8. Gene set enrichment analyses

Table S9. Important edges for UC, CAD, and SCZ

Table S10. Expected and observed frequencies of important edge types in the neighborhood of core genes

Table S11. Important metabolic connections in CAD

Table S13. Comparing signal propagated from HSPs to core genes and peripherals

Table S14. Individual statistics for knockdown perturbagens

Table S15. Individual statistics for overexpression perturbagens

Table S16. Individual overlap indices and empiric *p* values (FDR adjusted) of perturbagens across traits and cell types

Table S17. Model parameters for co-perturbation simulations of individual gene combinations

Table S18. Chi-squared test statistics for GEARS co-perturbation simulations

Document S2. Article plus supplemental information
