# Supplementary material for: Exploring the omnigenic architecture of selected complex traits
Source: Am J Hum Genet. 2025 Aug 4;112(9):2115–37. doi: 10.1016/j.ajhg.2025.07.006 (PMC12461020; doi:10.1016/j.ajhg.2025.07.006)
Supplement: Document S1. Figures S1–S15, Table S12, and Notes S1–S3 [file mmc1.pdf]

The American Journal of Human Genetics, Volume *112*

Supplemental information

Exploring the omnigenic architecture of selected complex traits

Florin Ratajczak, Matthias Heinig, and Pascal Falter-Braun

SUPPLEMENTAL NOTES

Note S1: CAD and SCZ Core Genes

We gathered 209 genes causative for Mendelian disorders with phenotypic similarity to CAD by querying OMIM for established standardized phenotype terms. Speos returned a total of 1381 candidate core genes with varying degrees of confidence (Figure S4a). OMIM-derived genes and candidates are enriched for mouse knockout genes and differentially expressed genes[4] (Figure S1a). We used 642 core genes (CS11 + OMIM-derived genes) and 16014 peripherals (CS0) for subsequent analyses. For schizophrenia (SCZ), we gathered 110 genes causative for Mendelian disorders with phenotypic similarity to SCZ by querying OMIM for established standardized phenotype terms. Speos returned a total of 2037 candidate core genes with varying degrees of confidence (Figure S4c). OMIM-derived genes and candidates are enriched for mouse knockout genes and differentially expressed genes (Figure S4c). We used a set of 321 core genes (CS11 + OMIM-derived genes) and 15293 peripheral genes for subsequent analyses.

Note S2: Network Proximity of Core Genes and HSPs

The omnigenic model assumes that a subset of peripheral genes have a high GWAS signal due to an extraordinary position in the gene-gene graph relative to core genes[5,6]. Given accurate edge weights, this position can be modeled using label propagation[7,8]. For this analysis a distribution is initialized so that for every HSP and 0 for every other gene. These values are then propagated along the direction of edges in a graph and aggregated using a nodes previous value and the mean of the neighborhood to obtain (Figure S6a). Finally, if the propagated values are higher in core genes than in peripherals, then core genes are collectively closer to HSPs than peripherals. We used our multi-modal graph to investigate if signal propagated from HSPs along directed edges reaches coregenes with a higher magnitude than other genes. When comparing the distribution of core genes to those of peripherals we observed a significant difference only after 5 iterations of label propagation (Figure S6b). However, when reversing the direction of propagation along the edges, core genes received a significantly higher median than peripherals after 1, 3 and 5 iterations, indicating that core genes are significantly closer than peripherals

"upstream" of HSPs (Figure S6c). We compared these results to initializations of using HSPs of other traits and randomly selected genes and found that UC core genes were not significantly closer to UC HSPs than expected by chance (empiric $p<0.05$, Figure S3d). However, most randomly selected gene sets were closer to any set of core genes than to other peripherals. We also tested an alternative approach where we used edge weights corresponding to the trait-specific edge importance (Figure 6a, Figure S6e). Again, most genes are close to core genes but HSPs are not significantly closer than randomly selected genes (Figure S6e). We therefore conclude that core genes are generally central in genetic networks.

Note S3: RA and AD Core Genes

We gathered 279 genes causative for Mendelian disorders with phenotypic similarity to rheumatoid arthritis (RA) by querying OMIM for established standardized phenotype terms. Speos returned a total of 1528 candidate core genes with varying degrees of confidence (Figure S11a). OMIM-derived genes and candidates are enriched for mouse knockout genes and differentially expressed genes[9] (Figure S11a). We used 658 core genes (CS11 + OMIM-derived genes) and 15577 peripherals (CS0) for subsequent analyses. For Alzheimer's disease (AD), we gathered 115 genes causative for Mendelian disorders with phenotypic similarity by querying OMIM for established standardized phenotype terms. Speos returned a total of 2035 candidate genes with varying degrees of confidence (Figure S11d). OMIM-derived genes and candidates are enriched for mouse knockout genes and differentially expressed genes, using the top 1000 up- and downregulated genes computed across 22 large-

scale gene expression datasets[10] (Figure S11). We used 658 core genes (CS11 + OMIM-derived genes) and 15577 peripherals (CS0) for subsequent analyses.

SUPPLEMENTAL FIGURES

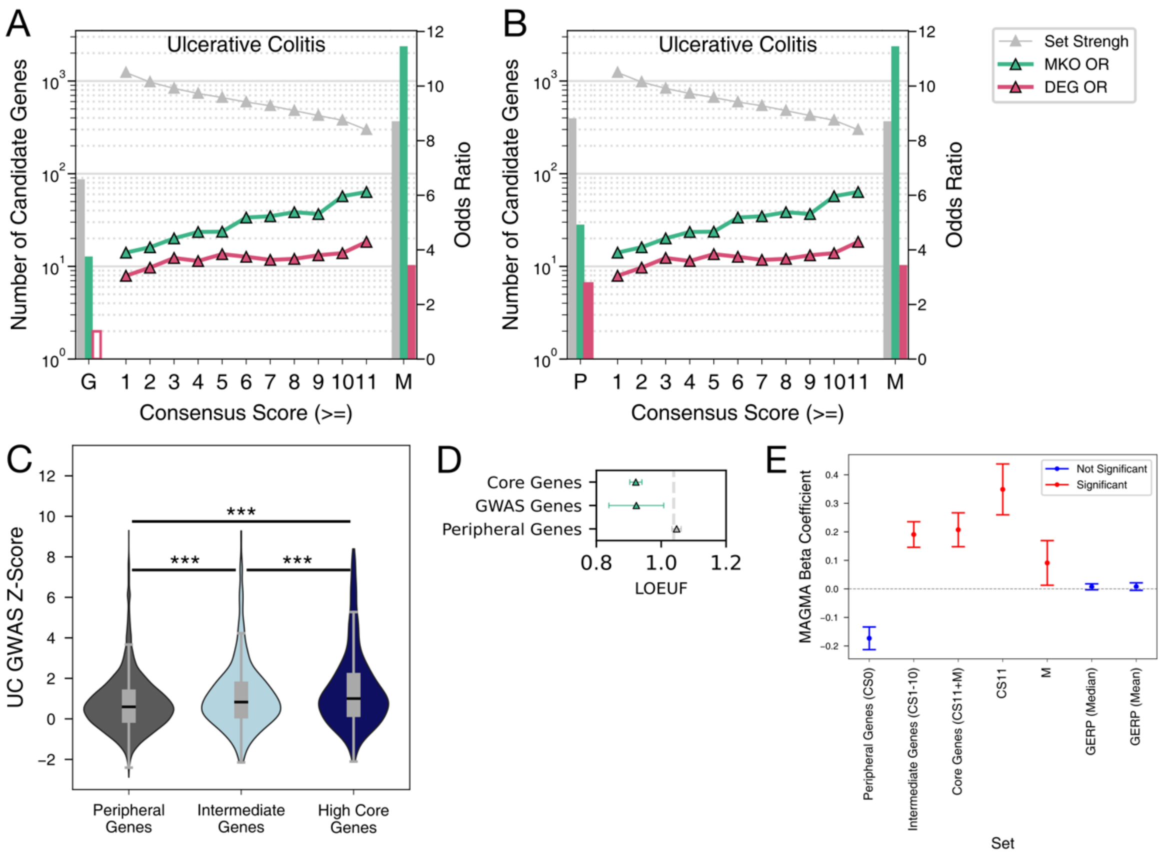

Figure S1: Extended external validation of core gene candidates for ulcerative colitis. A Set strength (grey, left y-axis) of GWAS genes (G), Speos Candidates (Consensus Score 1-11) and Mendelian disorder Genes (M) for coronary artery disease. Teal and red indicate odds ratios (OR, right y-axis) of mouse knockout (MKO) and differentially expressed genes (DEG) among the indicated sets, respectively. Markers and filled bars indicate a significant enrichment compared to peripheral genes (FDR < 0.05, individual p-values in Table S3). B Analogous to A, but now the left bars (P) denote set strength (grey, left y-axis) and ORs (teal and red, right y-axis) of novel candidate core genes among the top 500 genes prioritized by the polygenic priority score (PoPs) method. C UC GWAS Z-score of peripheral genes, ambivalent genes and core genes based on a recent large-scale GWAS (GCST90446794). Asterisks denote significant differences (two-sided t-test, p<0.001). D 95% confidence intervals (CI) of loss of function observed/expected upper bound fraction (LOEUF) among core genes and GWAS genes for ulcerative colitis. Colored CIs indicate significant differences to peripheral genes (Tukey's HSD, p<0.05, see S6 for individual p-values) E Beta coefficients and 95% confidence intervals of selected gene sets using the MAGMA gene set analysis based on a linear mixed model. Groups are selected by Consensus Score (CS) or OMIM-derived genes (M). MAGMA fits individual models for each gene set. Significance is tested one-sided (greater than 0) for gene sets and two-sided for covariates (gene-level median and mean genomic evolutionary rate profiling (GERP)

scores[1] across all exons. GERP scores are originally calculated across 91 mammalian species). Additional internal covariates are linkage disequilibrium, gene size, gene density, inverse minor allele count and the logarithms thereof.

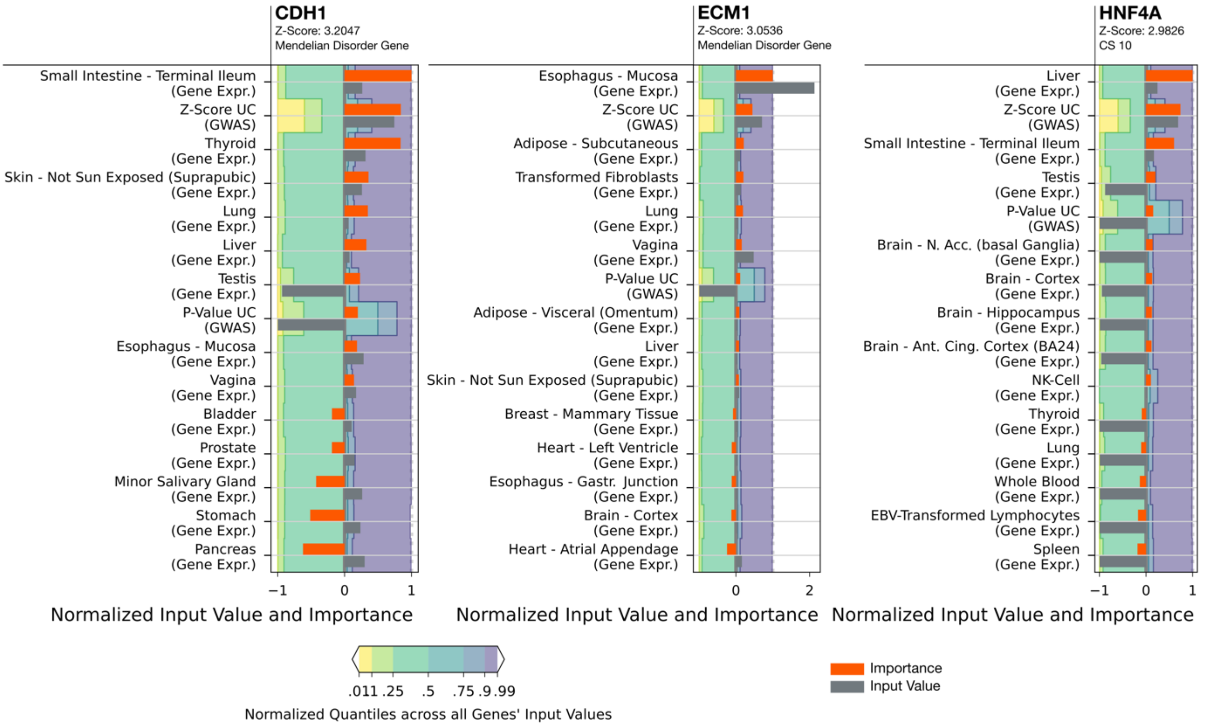

Figure S2: Feature importance scores for selected genes. Feature importance scores (orange bars) and input values for each feature (grey bars). Positive importance scores indicate arguments in favor of core gene prediction while negative scores speak against core gene prediction. Shown are the ten features with the highest and five features with the lowest score.

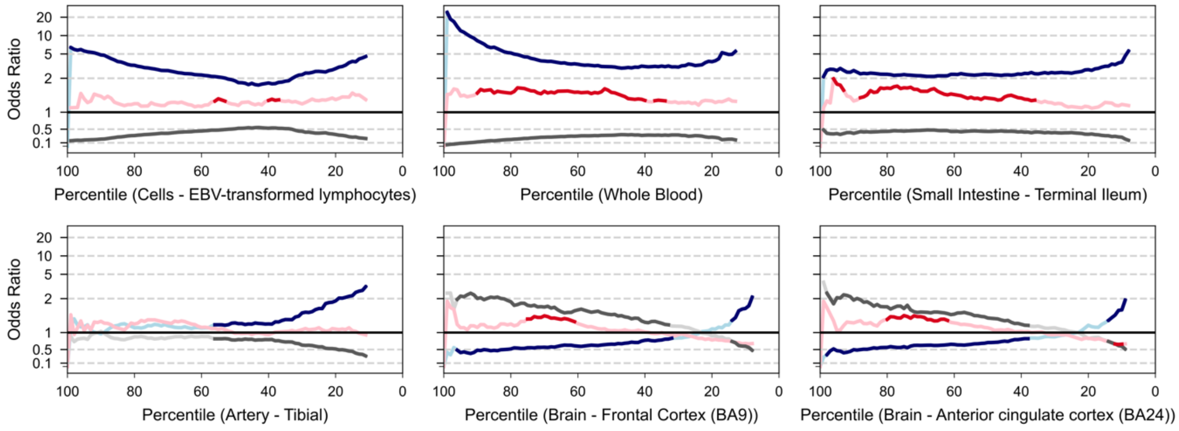

Figure S3: Enrichment of core genes, HSPs and peripherals in selected tissues. Odds ratios of core genes (blue), HSPs (red) and peripherals (grey) in or above a given percentile of the genome after sorting genes according to their expression in the respective tissue or cell type (x-axis). Darker colors indicate significant enrichment/depletion

(Fisher's exact test, FDR < 0.05, individual p-values s. Table S6). HSPs are selected based on a GWAS Z-Score larger than 5 calculated from a recent large-scale ulcerative colitis GWAS (GCST90446794).

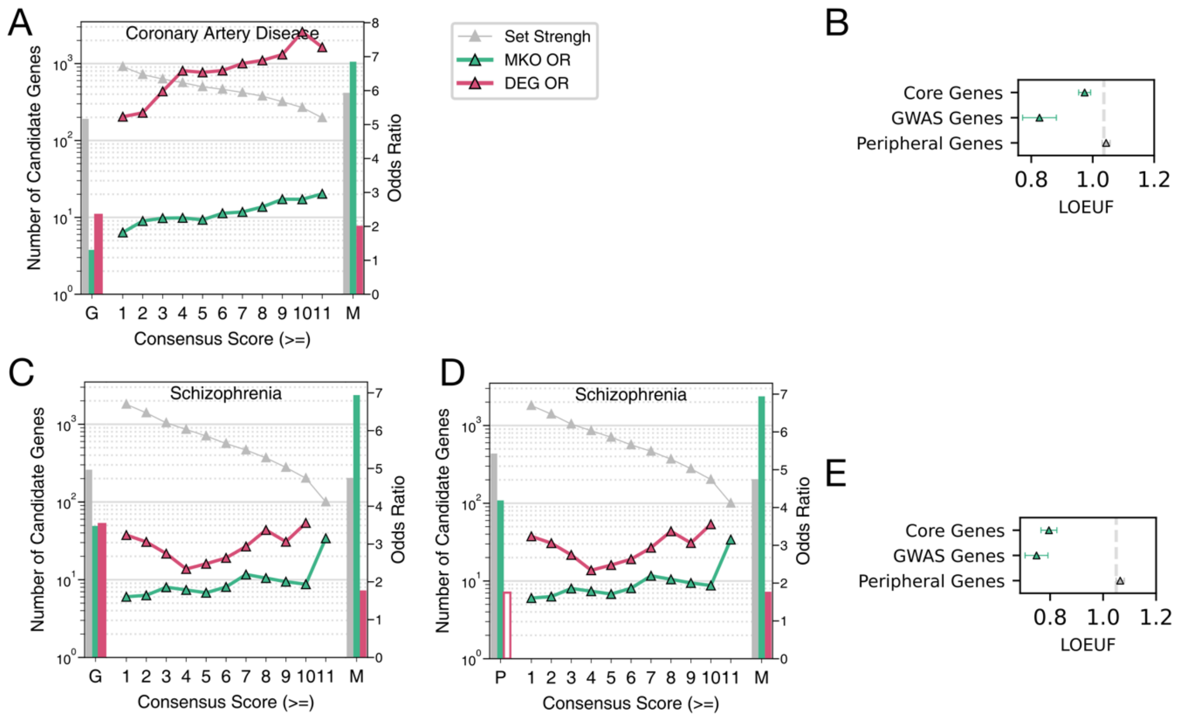

Figure S4: External validation of core gene candidates for coronary artery disease and schizophrenia. A Set strengths (grey, left y-axis) of GWAS genes (G), Speos Candidates (Consensus Score 1-11) and Mendelian disorder Genes (M) for coronary artery disease. Teal and red indicate odds ratios (OR, right y-axis) of mouse knockout (MKO) and differentially expressed genes (DEG) among the indicated sets, respectively. Markers and filled bars indicate a significant enrichment compared to peripheral genes (FDR < 0.05, individual p-values in Table S3). B 95% confidence intervals (CI) of loss of function observed/expected upper bound fraction (LOEUF) among core genes and GWAS genes. Colored CIs indicate significant differences to periphreal genes (Tukey's HSD, p<0.05, see Table S6 for individual p-values) C Validation of schizophrenia core and GWAS genes, analogous to A. D Analogous to C, but now the left bars (P) denote set strength (grey, left y-axis) and ORs (teal and red, right y-axis) of novel candidate core genes among the top 500 genes prioritized by the polygenic priority score (PoPs)

method. E Validation of LOEUF constraint among core genes and GWAS genes for schizophrenia, analogous to B.

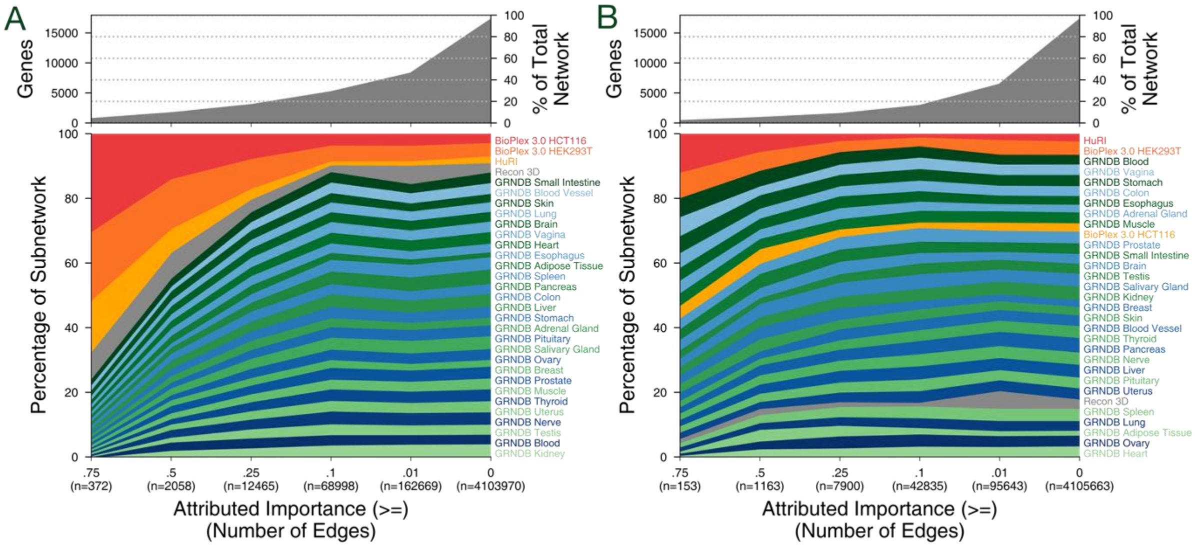

Figure S5: Distribution of edge types among edges above certain thresholds of attributed importance for coronary artery disease and schizophrenia. Edge types are sorted by their distribution at >= .75 attributed importance. The grey graph above shows how many genes are still part of the network when the thresholds are applied. A Distribution for coronary artery disease. B Distribution for schizophrenia.

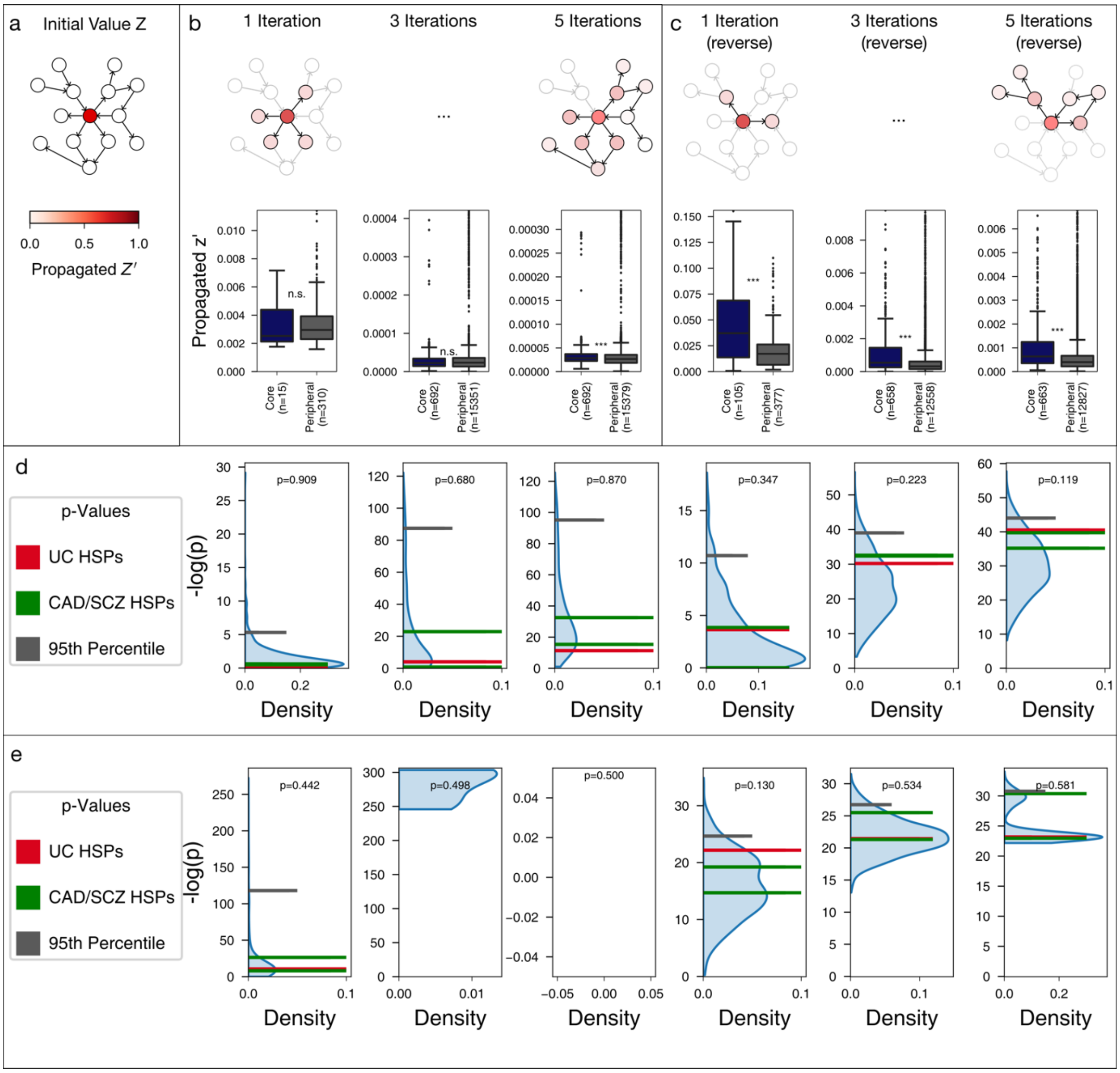

Figure S6: Label propagation analyses. a in the initial state, HSPs are initialized with a value z=1 while all other genes are initialized with z=0. B Z is propagated along the edges of the graph for 1, 3 or 5 iterations and the resulting distribution of Z' of core genes is significantly higher than that of peripheral genes (t-test, FDR < 0.05, for individual p-values see Table S10). c Reversing the direction of all edges and propagating Z also leads to a significantly higher Z' of core genes compared with peripheral genes (t-test, FDR < 0.05, for individual p-values see Table S10). D When drawing 100 random sets of peripheral genes, HSPs do not lead to significantly higher Z' of core genes compared to peripheral genes (empiric p > 0.05, for individual p-values see Table S10). e Repeating the experiments from d, but with the trait-specific edge weights (Figure 3a), leads to similar results. (empiric p > 0.05, for individual p-values see Table S10).

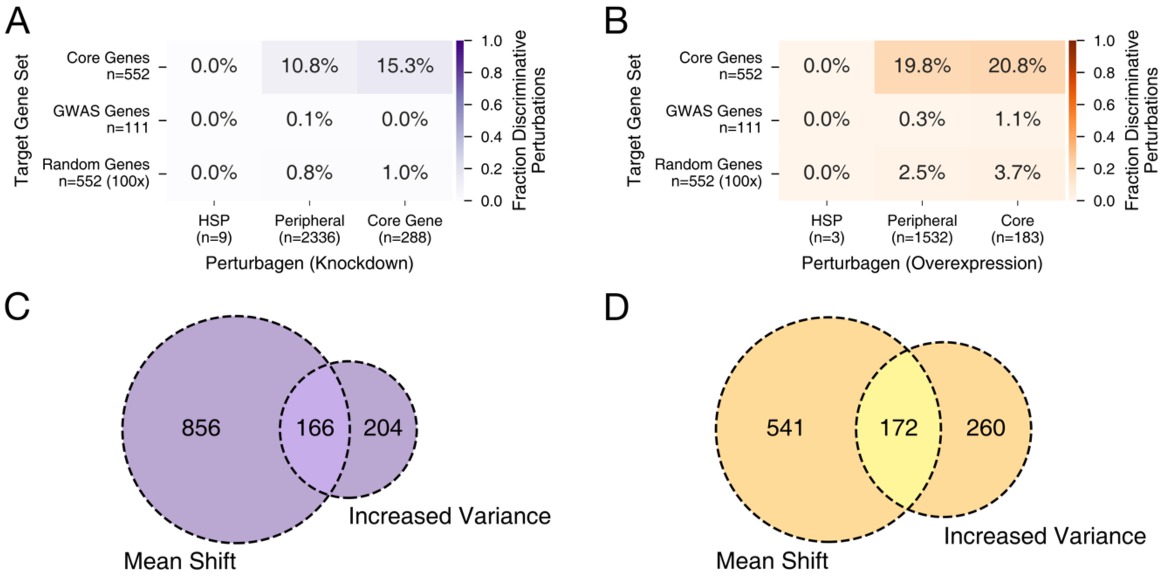

Figure S7: Perturbagens leading to a significant increase in variance for core genes of ulcerative colitis (UC). Fraction of significant knockdown (A) and overexpression (B) perturbagens for UC core genes in HT29 cells contrasted with the fraction of significant perturbagens for GWAS genes (middle row) randomly selected genes (lower row, 100 repetitions) (one-sided F-test, FDR < 0.05, individual p-values in Table S13). C: Differences and intersection of genes leading to a significant discriminative perturbation of UC core genes by mean shift (Figure 6B) and increase in variance (Figure S9A) in knockdown experiments. D: Differences and intersection of genes leading to a significant discriminative perturbation by mean shift (Figure 6C) and increase in variance (Figure S9B) in overexpression experiments.

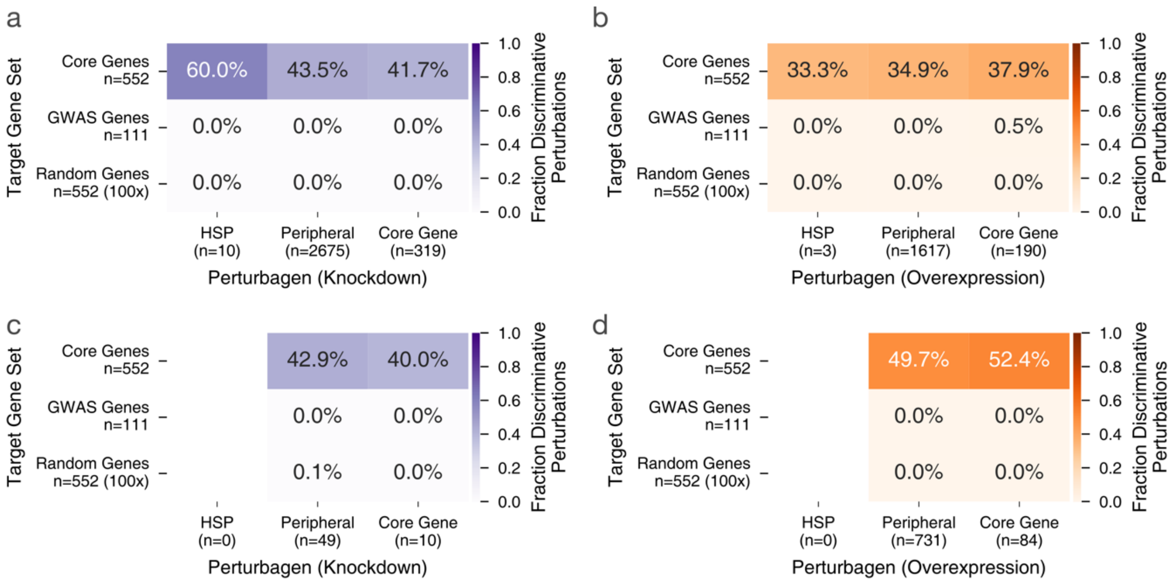

Figure S8: Significant discriminative perturbagens for ulcerative colitis. Fraction of significant knockdown (a,c) and overexpression (b, d) perturbagens for UC core genes in PC3 (a, b) and HEK203T (c, d) cells contrasted with the fraction of significant perturbagens for GWAS genes (middle row) randomly selected genes (lower row, 100 repetitions) (t-test, FDR < 0.05, individual p-values in Table S14).

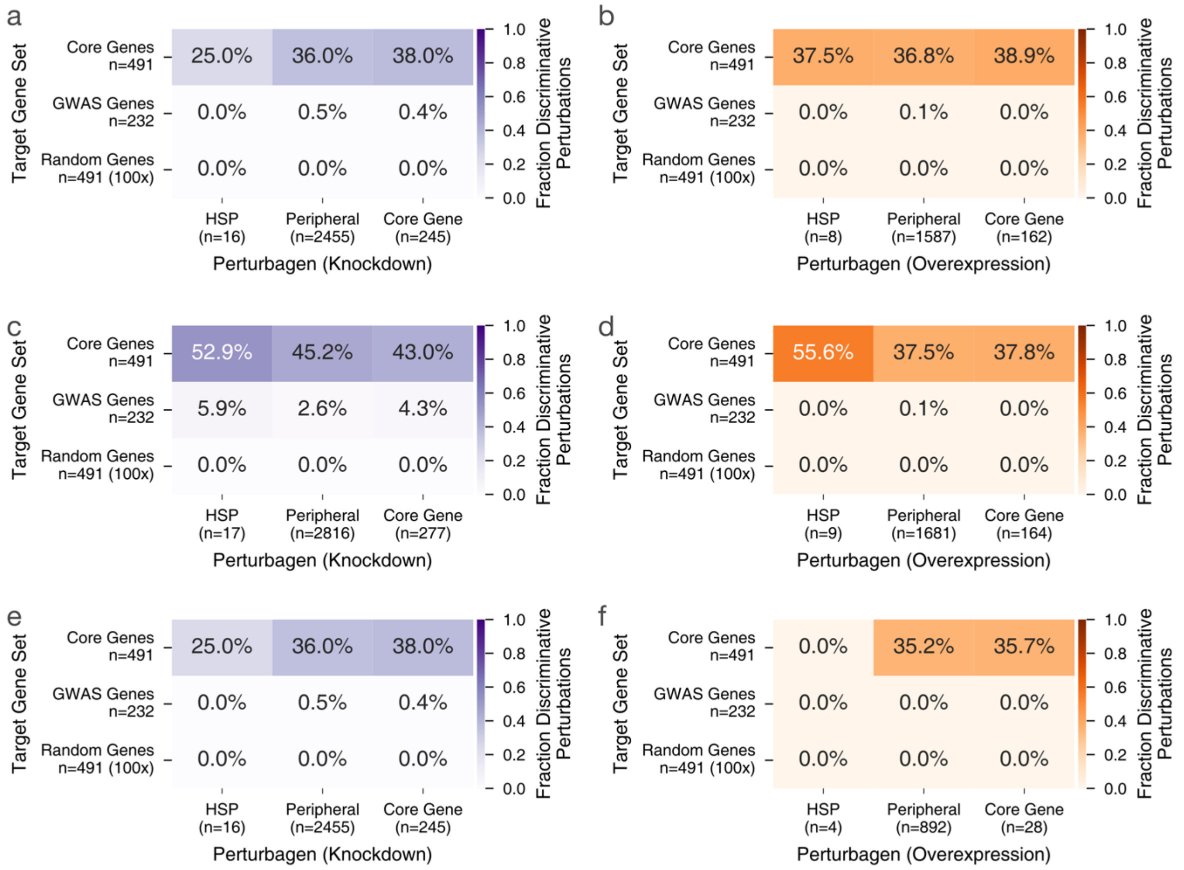

Figure S9: Significant discriminative perturbagens for coronary artery disease. Fraction of significant knockdown (a,c,e) and overexpression (b, d,f) perturbagens for CAD core genes in HT29 (a,b), PC3 (c, d) and HEK203T (e, f)

cells contrasted with the fraction of significant perturbagens for GWAS genes (middle row) randomly selected genes (lower row, 100 repetitions) (t-test, FDR < 0.05, individual p-values in Table S14)

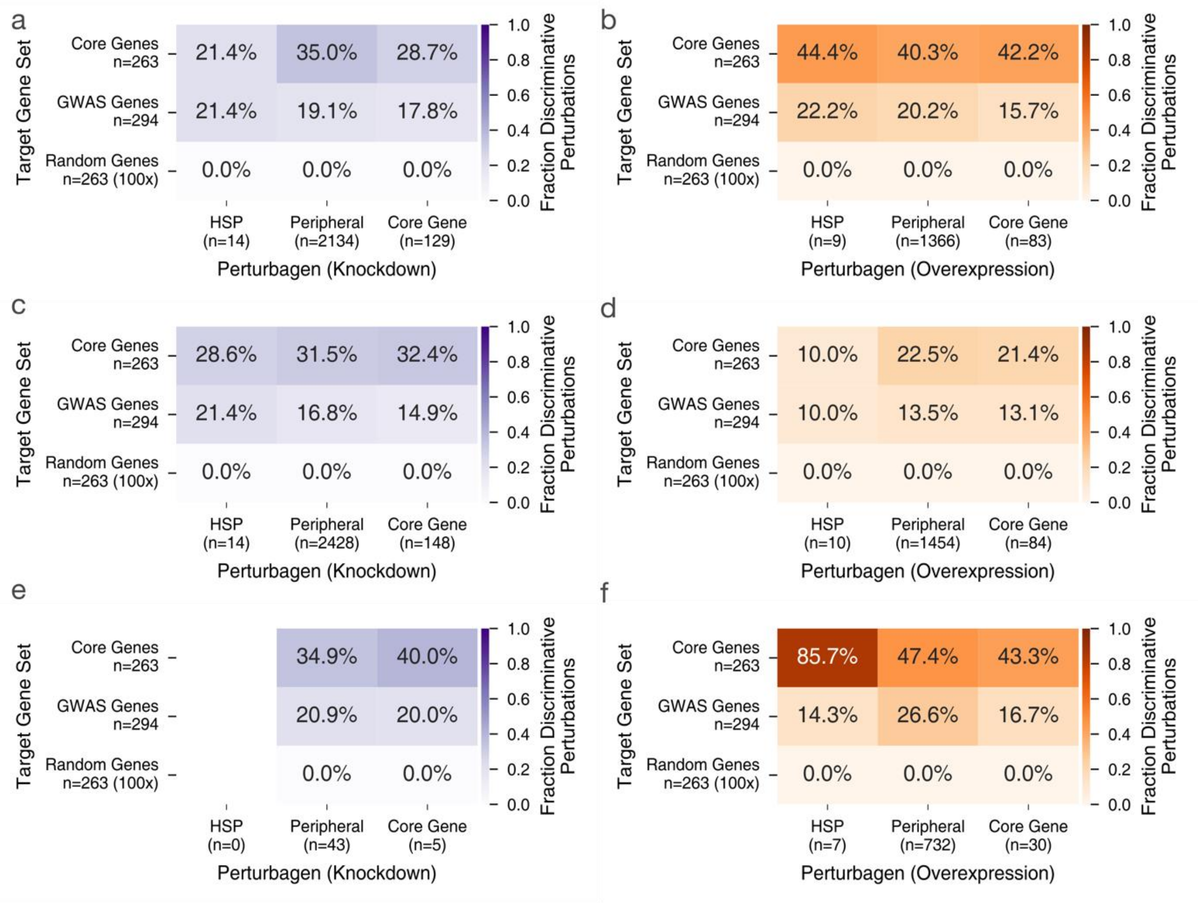

Figure S10: Significant discriminative perturbagens for schizophrenia. Fraction of significant knockdown (a,c,e) and overexpression (b,d,f) perturbagens for SCZ core genes in HT29 (a,b), PC3 (c, d) and HEK203T (e, f) cells

contrasted with the fraction of significant perturbagens for GWAS genes (middle row) randomly selected genes (lower row, 100 repetitions) (t-test, FDR < 0.05, individual p-values in Table S14)

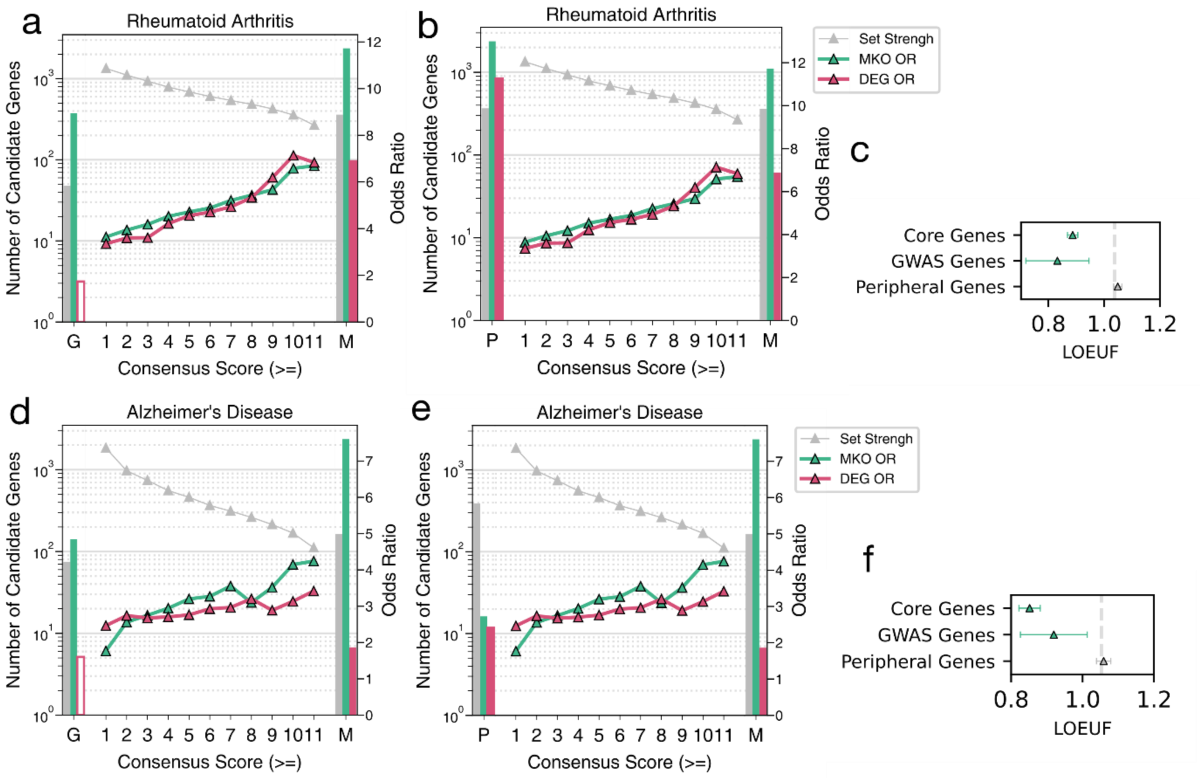

Figure S11: External validation of core gene candidates for rheumatoid arthritis and Alzheimer's disease. a Set strengths (grey, left y-axis) of GWAS genes (G), Speos Candidates (Consensus Score 1-11) and Mendelian disorder Genes (M) for coronary artery disease. Teal and red indicate odds ratios (OR, right y-axis) of mouse knockout (MKO) and differentially expressed genes (DEG) among the indicated sets, respectively. Markers and filled bars indicate a significant enrichment compared to peripheral genes (FDR < 0.05, individual p-values in Table S3). b d Analogous to c, but now the left bars (P) denote set strength (grey, left y-axis) and ORs (teal and red, right y-axis) of novel candidate core genes among the top 500 genes prioritized by the polygenic priority score (PoPs) method. c 95% confidence intervals (CI) of loss of function observed/expected upper bound fraction (LOEUF) among core genes and GWAS genes for rheumatoid arthritis. Colored CIs indicate significant differences to peripheral genes (Tukey's HSD, p<0.05, see Table S6 for individual p-values) d Validation of Alzheimer's disease core and GWAS genes, analogous to a. e Validation of Alzheimer's disease core and PoPs prioritized genes, analogous to b. f Validation of LOEUF constraint among core genes and GWAS genes for schizophrenia, analogous to c.

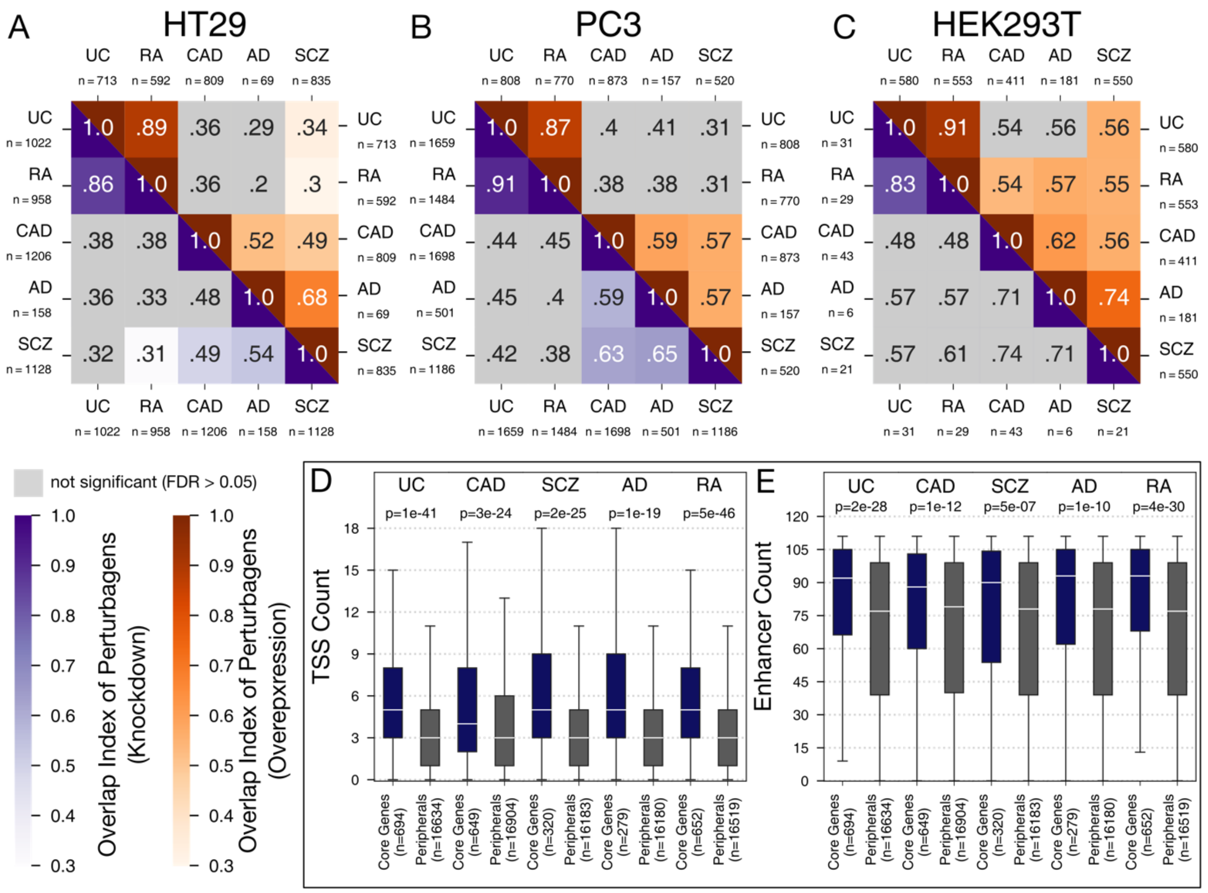

Figure S12: Regulatory complexity of core gene sets. A-C Overlap index of discriminative perturbagens across traits in HT29, PC3 and HEK293T cells. The upper triangle reflects overlap indices for overexpression perturbagens and the lower triangle reflects overlap indices for knockdown perturbagens. Overlap indices that are significantly larger than expected by chance are colored (empirical FDR < 0.05, individual p-values in Table S16), else grey. D Count of promoters (TSS, transcription start sites) per gene. Traits are annotated with an unadjusted p-value of two-sided U-tests. C Count of active enhancer elements across biosamples per Gene. Traits are annotated with an unadjusted p-value of two-sided U-tests.

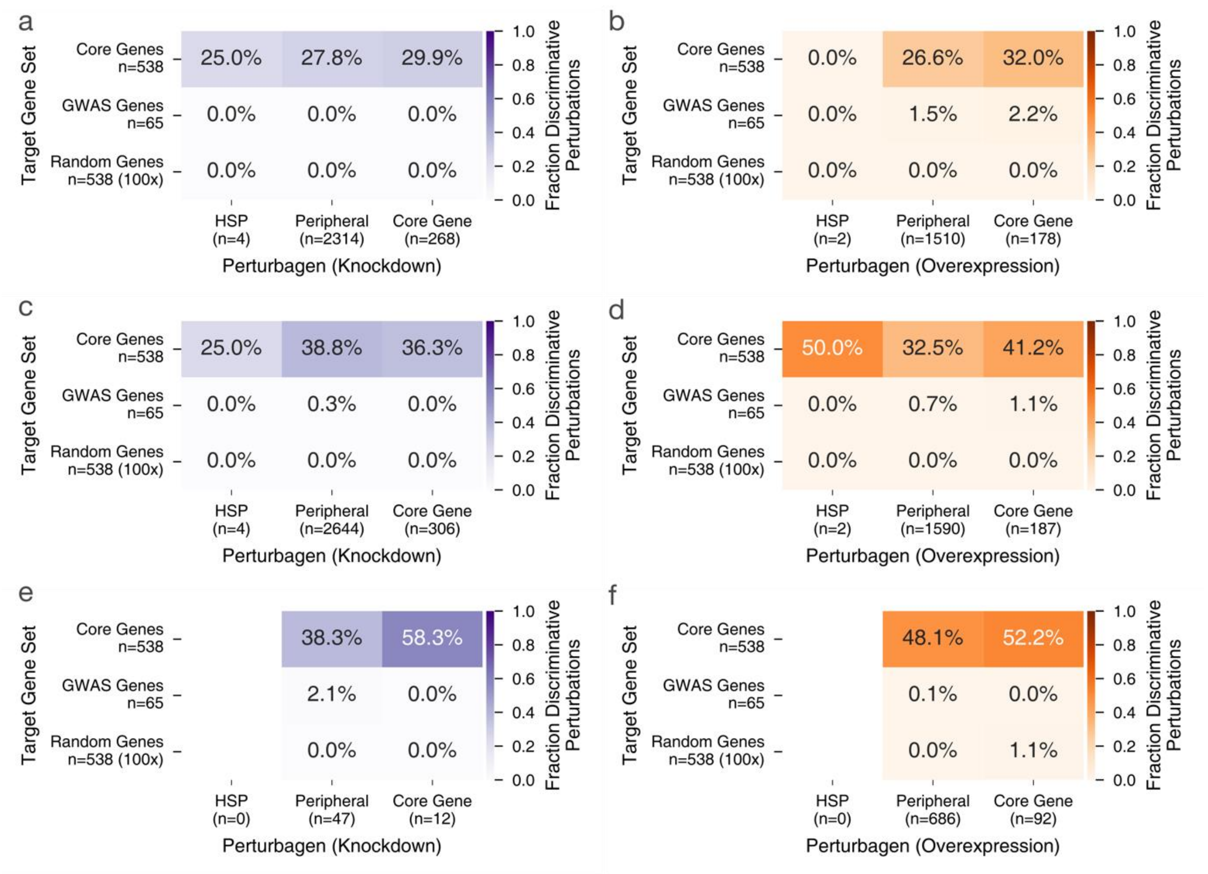

Figure S13: Significant discriminative perturbagens for rheumatoid arthritis. Fraction of significant knockdown (a,c,e) and overexpression (b,d,f) perturbagens for RA core genes in HT29 (a,b), PC3 (c, d) and HEK203T (e, f) cells contrasted with the fraction of significant perturbagens for GWAS genes (middle row) randomly selected genes (lower row, 100 repetitions) (t-test, FDR < 0.05, individual p-values in Table S14)

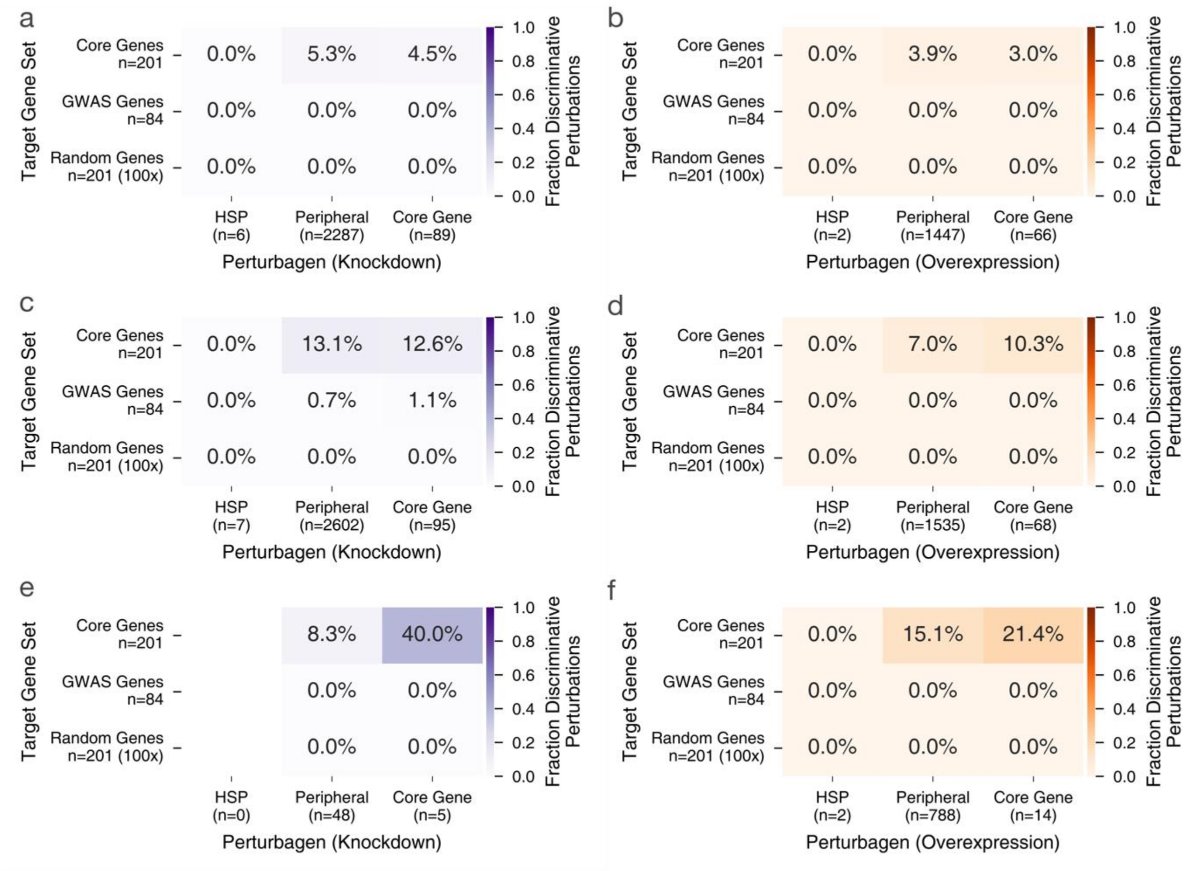

Figure S14: Significant discriminative perturbagens for Alzheimer's disease. Fraction of significant knockdown (a,c,e) and overexpression (b,d,f) perturbagens for AD core genes in HT29 (a,b), PC3 (c, d) and HEK203T (e, f) cells contrasted with the fraction of significant perturbagens for GWAS genes (middle row) randomly selected genes (lower row, 100 repetitions) (t-test, FDR < 0.05, individual p-values in Table S14)

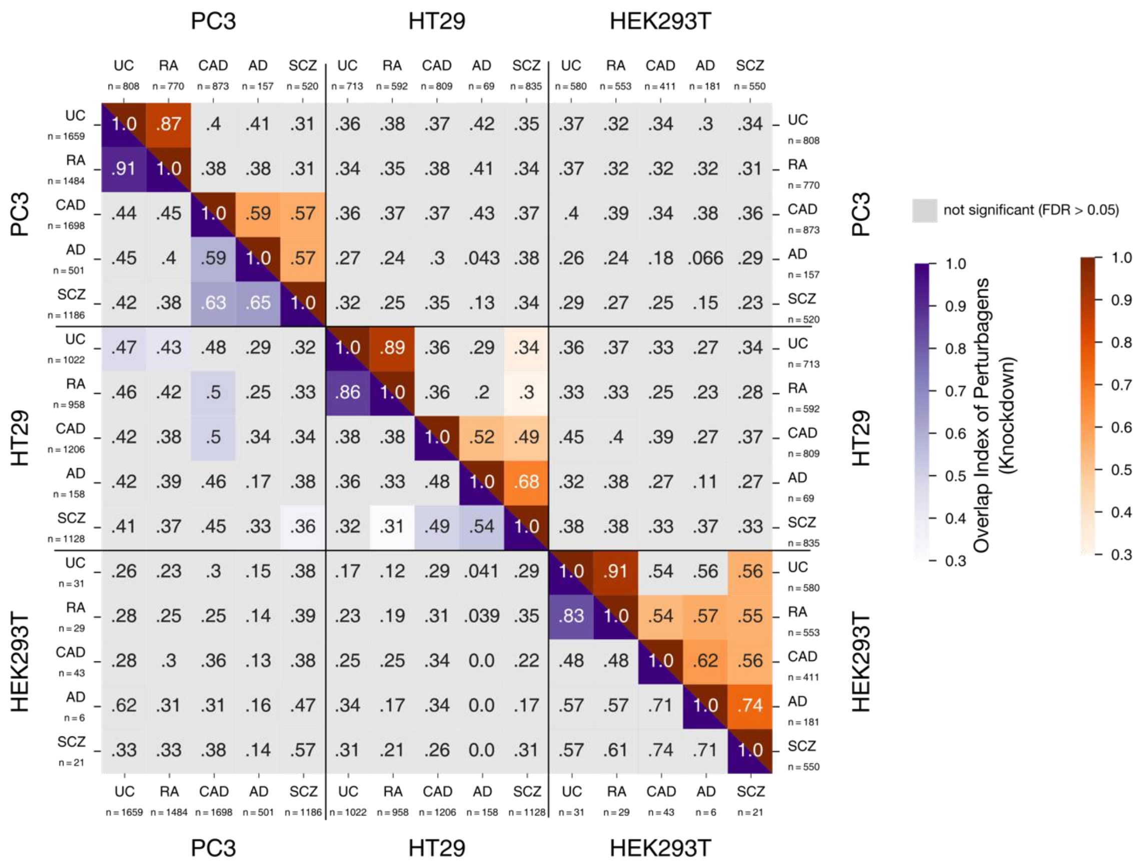

Figure S15: Overlap index of discriminative perturbagens across traits in three cell types. The upper triangle reflects overlap indices for overexpression perturbagens and the lower triangle reflects overlap indices for knockdown perturbagens. Overlap indices that are significantly larger than expected by chance are colored (empiric FDR < 0.05, individual p-values in Table S16), else grey.

SUPPLEMENTAL TABLES

SNP rsid	p-value	HSP gene	Source GWAS
rs3766606	4e-9	*PARK7*	[2]
rs7554511, rs35730213	7e-31, 2e-24	*INAVA*	[2], [3]
rs395157, rs395157	9e-12, 2e-10	*OSMR*	[2], [3]
rs4246905	1e-15	*TNFSF15*	[2]
rs483905	3e-10	*MAML2*	[2]
rs661054	3e-20	*NXPE1*	[2]
rs8005161	3e-9	*GPR65*	[2]
rs7404095, rs59790099	2e-8, 2e-11	*PRKCB*	[2], [3]
rs17780256	6e-13	*SLC39A11*	[2]
rs2124440	1e-8	*ITGA4*	[3]
rs3776414	3e-8	*DAP*	[3]
rs55982276	9e-9	*PHYKPL*	[3]
rs2721933	2e-8	*TRPS1*	[3]
rs2790211	2e-9	*IPMK*	[3]
rs11066188	2e-8	*HECTD4*	[3]
rs4462528	1e-13	*GALC*	[3]
rs71407313	2e-11	*CRTC3*	[3]

Table S12: Mapping of significant UC SNPs to the respective HSP genes. GWAS are identified by their literature references.

SUPPLEMENTAL REFERENCES

1. Davydov, E. V. *et al.* Identifying a High Fraction of the Human Genome to be under Selective Constraint Using GERP++. *PLoS Comput. Biol.* **6**, e1001025 (2010).
2. Liu, J. Z. *et al.* Association analyses identify 38 susceptibility loci for inflammatory bowel disease and highlight shared genetic risk across populations. *Nat. Genet.* **47**, 979–986 (2015).
3. Liu, Z. *et al.* Genetic architecture of the inflammatory bowel diseases across East Asian and European ancestries. *Nat. Genet.* **55**, 796–806 (2023).
4. Balashanmugam, M. V., Shivanandappa, T. B., Nagarethinam, S., Vastrad, B. & Vastrad, C. Analysis of Differentially Expressed Genes in Coronary Artery Disease by Integrated Microarray Analysis. *Biomolecules* **10**, (2020).
5. Boyle, E. A., Li, Y. I. & Pritchard, J. K. An Expanded View of Complex Traits: From Polygenic to Omnigenic. *Cell* **169**, 1177–1186 (2017).
6. Liu, X., Li, Y. I. & Pritchard, J. K. Trans Effects on Gene Expression Can Drive Omnigenic Inheritance. *Cell* **177**, 1022-1034.e6 (2019).
7. Zhu, Xiaojin & Ghahramani, Zoubin, (last). Learning from Labeled and Unlabeled Data with Label Propagation. Preprint at https://mlg.eng.cam.ac.uk/zoubin/papers/CMU-CALD-02-107.pdf (2002).
8. Huang, Q., He, H., Singh, A., Lim, S.-N. & Benson, A. R. Combining Label Propagation and Simple Models out-performs Graph Neural Networks. in *9th International Conference on Learning Representations, ICLR 2021, Virtual Event, Austria, May 3-7, 2021* (OpenReview.net, 2021).

9. Zhang, D. *et al.* Identification of differentially expressed and methylated genes associated with rheumatoid arthritis based on network. *Autoimmunity* **53**, 303–313 (2020).

10. Hill, M. A. & Gammie, S. C. Alzheimer's disease large-scale gene expression portrait identifies exercise as the top theoretical treatment. *Sci. Rep.* **12**, 17189 (2022).
